# Pan-Cancer Mutational and Transcriptional Analysis of the Integrator Complex

**DOI:** 10.3390/ijms18050936

**Published:** 2017-04-29

**Authors:** Antonio Federico, Monica Rienzo, Ciro Abbondanza, Valerio Costa, Alfredo Ciccodicola, Amelia Casamassimi

**Affiliations:** 1 Institute of Genetics and Biophysics “Adriano Buzzati Traverso”, CNR, 80131 Naples, Italy; antonio.federico@igb.cnr.it (A.F.); valerio.costa@igb.cnr.it (V.C.); alfredo.ciccodicola@igb.cnr.it (A.C.); 2 Department of Science and Technology, University of Naples “Parthenope”, 80143 Naples, Italy; 3 Department of Environmental, Biological, and Pharmaceutical Sciences and Technologies, University of Campania “Luigi Vanvitelli”, 81100 Caserta, Italy; monica.rienzo@unicampania.it; 4 Department of Biochemistry, Biophysics and General Pathology, University of Campania “Luigi Vanvitelli”, Via L. De Crecchio, 80138 Naples, Italy; ciro.abbondanza@unicampania.it

**Keywords:** integrator complex, somatic mutations, transcriptome profiling, human cancers, TCGA data analysis

## Abstract

The integrator complex has been recently identified as a key regulator of RNA Polymerase II-mediated transcription, with many functions including the processing of small nuclear RNAs, the pause-release and elongation of polymerase during the transcription of protein coding genes, and the biogenesis of enhancer derived transcripts. Moreover, some of its components also play a role in genome maintenance. Thus, it is reasonable to hypothesize that their functional impairment or altered expression can contribute to malignancies. Indeed, several studies have described the mutations or transcriptional alteration of some Integrator genes in different cancers. Here, to draw a comprehensive pan-cancer picture of the genomic and transcriptomic alterations for the members of the complex, we reanalyzed public data from The Cancer Genome Atlas. Somatic mutations affecting Integrator subunit genes and their transcriptional profiles have been investigated in about 11,000 patients and 31 tumor types. A general heterogeneity in the mutation frequencies was observed, mostly depending on tumor type. Despite the fact that we could not establish them as cancer drivers, *INTS7* and *INTS8* genes were highly mutated in specific cancers. A transcriptome analysis of paired (normal and tumor) samples revealed that the transcription of *INTS7*, *INTS8*, and *INTS13* is significantly altered in several cancers. Experimental validation performed on primary tumors confirmed these findings.

## 1. Introduction

The integrator complex (INT) is one of the major components of the RNA polymerase II mediated transcription machinery, playing a role in the regulation of most dependent genes [1,2,3]. This multiprotein consists of at least 14 different subunits, even though its structure and composition have not yet been fully characterized [3]. It was originally discovered as a complex implicated in the 3′-end formation of noncoding uridine-rich small nuclear RNAs [1,4,5,6,7,8]. However, in the last few years, many studies have promptly indicated broader functions for this complex, extending its role to other aspects of transcriptional regulation [2,3]. For instance, several experimental studies have also allowed for the assumption of a critical role for the INT complex in the activation of protein-coding genes, particularly in the pause-release and elongation of polymerase [9,10,11]. Moreover, in a very recent paper, the INT complex was shown to mediate the biogenesis of transcripts derived from distal regulatory elements (enhancers) involved in the tissue- and temporal-specific regulation of gene expression in metazoans [12]. Finally, some of its components (particularly IntS3, IntS6, and IntS7) were shown to participate, together with nucleic acid binding proteins (NABPs), in the formation of other protein complexes involved in DNA and RNA metabolism, including the DNA damage response [2,13,14,15,16].

It is worth noting that, given its main role in transcription regulation and nucleic acid metabolism, it is feasible that some INT subunits are also involved in human cancer [2]. Indeed, *INTS6/DICE1* was earlier identified as a tumor suppressor gene in lung carcinomas where it was frequently downregulated [17,18], and in esophageal squamous cell carcinomas where mutations occurred, although at a low frequency [19]. Furthermore, promoter CpG hypermethylation and the downregulation of *INTS6/DICE1* expression was also observed in prostate cancer cells [20]. More recent studies have supported the notion that the *INTS6/DICE1* gene has a role in malignancy [21,22,23]. The involvement of this subunit in human malignancy can be hypothesized from its function in the DNA damage response and the maintenance of genome stability [24]. Similarly, *INTS3* was found to be significantly overexpressed in cell lines and tumor tissues from hepatocellular carcinoma (HCC) patients compared with their non-cancerous counterparts [25]. In the last few years, both microarray and exome sequencing analyses have suggested a possible role of *INTS8* in gastric cancer and peripheral T-cell lymphoma, respectively [26,27]. Furthermore, *INTS14/VWA9* was found to be upregulated in immortalized cells, cancer cells, and non-small-cell lung cancer tissues [28]. More recently, whole-exome sequencing revealed recurrent mutations in the *INTS2* gene of gastric cancer patients [29]. Finally, the promoter methylation of *INTS1*, enclosed in a panel with other genes, was used to discriminate with high sensitivity and specificity cervical intraepithelial neoplastic lesions (grade 2 or higher) from samples with no intraepithelial lesions or malignancy [30].

Overall, although most of these studies did not demonstrate a direct participation of INT subunits in carcinogenesis, it is expected that further alterations of these genes can be discovered in human cancer because of their key role in fundamental biological processes, often altered in malignancies.

Thus, to date, both mutations and altered expression have been reported for some subunits in specific cancer entities; however, a systematic and comprehensive approach deciphering the mutational status and the complete transcriptional profile of the whole INT complex across a large number of different cancer types is still lacking.

Here, The Cancer Genome Atlas (TCGA) deposited exome and RNA-Seq data [31] were used to perform a systematic analysis of both the mutational status and the transcriptional profile of all INT subunit genes across 31 distinct human cancer types.

## 2. Results

### 2.1. Mutational Profiling of Integrator Complex across Human Cancers

In order to systematically identify somatic mutations within genes encoding INT subunits, we analyzed exome-Seq data downloaded from TCGA (see Methods) for 31 cancer types. All of the INT subunit genes, *INTS6L* included, were analyzed*.* The number of samples for each cancer type is illustrated in Table 1.

Overall, 1916 point mutations and 128 insertions/deletions (both in-frame and frame-shift) affecting the protein products encoded by INT genes have been identified across all examined cancer types and all analyzed patients (>11,000). Silent (synonymous) mutations ranged between 19% (*INTS12*) and 42% (*INTS1*) of the total of detected mutations for each subunit. More than 50% of mutations detected in the TCGA data were non-synonymous. In particular, despite the highest percentage of silent mutations being identified in *INTS1*, we noticed that this gene has an overall huge number of mutations (528) and the largest number of non-synonymous ones (306).

**Figure 1:**
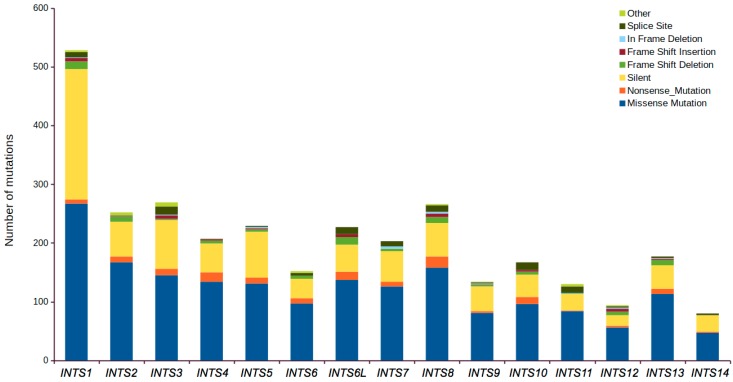
Stacked histograms showing the number of different classes of somatic mutations affecting INT genes as reported in the Mutation Annotation Files across all analyzed cancer entities.

Similarly, *INTS8* (209/266) and *INTS2* (193/252) were the other INT genes with high non-synonymous mutations. Nonsense mutations were more recurrent in *INTS8* and *INTS4* genes (19 and 16, respectively), whereas splice sites disrupting mutations were more frequently detected in *INTS3* and *INTS10* genes (14 and 13, respectively; Figure 1).

**Figure 2:**
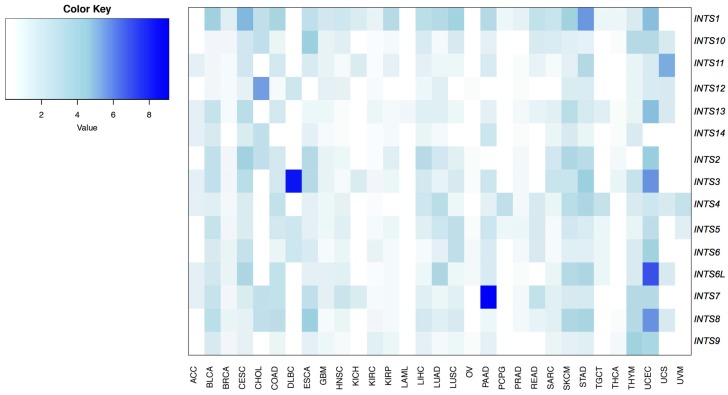
Frequency of patients carrying mutations in the INT subunits across the 31 analyzed tumors.

To measure the frequencies of somatic mutations for each INT gene across all tumor types, only non-synonymous mutations were considered. A global low mutation rate (from 0 to 9.09%) was found, with *INTS3* and *INTS7* being frequently mutated in lymphoid neoplasm diffuse large B-cell lymphoma (DLBC) (8.16%) and in pancreas adenocarcinoma (PAAD) (9.1%), respectively. Conversely, *INTS1* and *INTS4* were mutated in almost all cancer types, at low rates (Figure 2 and Table S1). *INTS9*, *INTS11, INTS13*, and *INTS14* were mutated in very few cancer types, with a very low mutation rate.

Distinguishing between so called “driver events”, i.e., somatic alterations (point mutations and gene rearrangements) that provide a growth advantage to cancer cells and which are positively selected during clonal tumor expansion, and mutations that stochastically occur during cancerogenesis (i.e., “passenger events”) is a great challenge. Mutations with a frequency higher than the background rate that tend to cluster in specific regions of protein-coding genes are likely to be driver genes. To assess whether members of the INT complex may be driver genes in a given cancer, we used OncodriveFML to analyze the pattern of somatic mutations undergoing positive selection, and therefore, those which are potentially involved in tumorigenesis [32].

**Figure 3:**
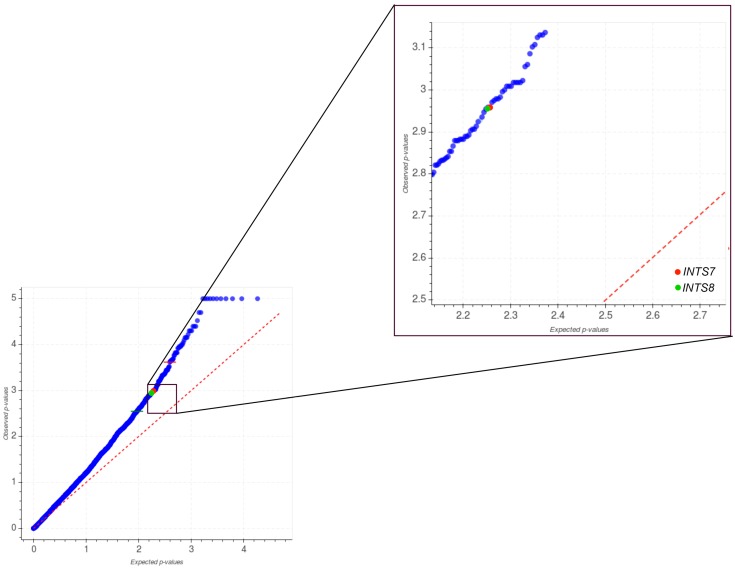
Quantile–quantile (QQ) plot comparing the expected and observed distribution of functional mutation (FM) bias *p*-values of genes detected in the UCEC cohort. Blue dots indicate genes reporting at least one somatic mutation in UCEC Exome-Seq data. Red dotted line indicates coincident values of expected and observed distributions of *p*-values. *INTS7* and *INTS8* genes are highlighted in red and green, respectively.

The *INTS7* and *INTS8* mutation patterns significantly differed from the background in uterine corpus endometrial carcinoma (UCEC) (*INTS7*, *q-val* = 0,182; *INTS8*, *q-val* = 0,184; Figure 3). However, when analyzing the co-occurrence among mutations in INT genes and those in known driver genes, we observed that a very small fraction of the total cohort of UCEC patients was hypermutated, and that most of them carried mutations in both *INTS7* and *INTS8* genes.

### 2.2. Differentially Expressed INT Subunits across Human Cancers

In order to ascertain whether the expression of INT genes is affected in human primary cancers, we took advantage of RNA-Seq datasets from paired samples (cancer vs. benign counterpart) available at the TCGA web portal. For the analysis of differential expression, we only considered, for each tumor type, samples with the corresponding “non-tumor” counterpart (see Methods and Table 1). Globally, 590 patients across 22 cancer types were analyzed. The gene expression profiles differed considerably between normal and tumor specimens, depending on the cancer type, as shown by the principal component analysis (see Supplementary_file_S1). The results of the gene expression profiling of INT genes across all available cancer types are summarized in Table S2.

**Figure 4:**
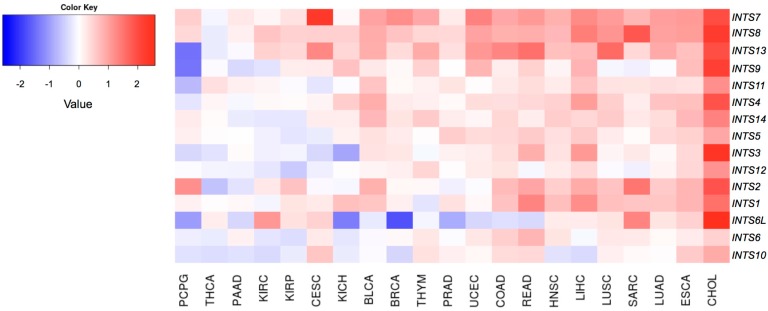
The heatmap shows the expression profiles of INT genes across analyzed cancer types.

Data indicate that a small subset of INT genes is consistently deregulated across several cancer types. In particular, a significant overexpression was measured for *INTS7, INTS8*, and *INTS13 (*Figure 4)*.* Notably, *INTS13*, which is significantly up-regulated in rectum adenocarcinoma, lung cancer small cells, and cholangiocarcinoma, is the most frequently deregulated INT gene at the transcriptional level (eight out of 22 analyzed cancer types).

On the other side, *INTS10*, *INTS6*, and *INTS6L* are more often downregulated across tumors. Our analysis reveals that among these genes, *INTS6L* is significantly down-modulated in breast cancer (logFC = −1.75; FDR = 1.47 × 10^−21^).

A strong deregulation of all INT genes was only measured in cholangiocarcinoma. Indeed, in this cancer type, 11/15 genes encoding INT subunits were overexpressed in tumor vs. healthy counterparts. Among them, the expression of *INTS6L* (logFC = 2.58; FDR = 1.26 × 10^−5^), *INTS3* (logFC = 2.42; FDR = 2.96 × 10^−10^), *INTS8* (logFC = 2.26; FDR = 9.84 × 10^−12^), *INTS9* (logFC = 2.18; FDR = 1.63 × 10^−7^), and *INTS7* (logFC = 1.97; FDR = 2.72 × 10^−9^) is significantly increased. Conversely, pheochromocytoma and paraganglioma (PCPG), thyroid cancer (THCA), and PAAD are the cancer types with the greatest number of downregulated genes.

### 2.3. The Expression of INTS7, INTS8 and INTS13 Is Increased in Human Primary Tumors

**Figure 5:**
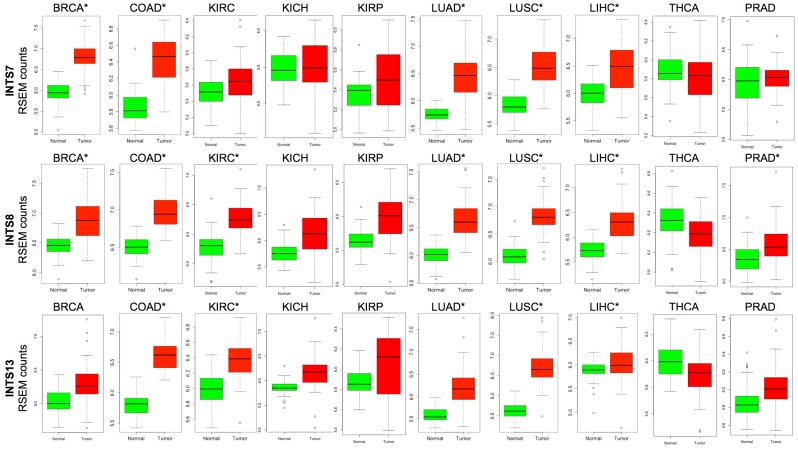
Boxplots showing the unbalanced expression of *INTS7*, *INTS8*, and *INTS13*, between tumors and normal counterparts. The asterisks indicate the tumor cohorts for which the deregulation has been validated in vitro.

The re-analysis of TCGA RNA-Seq data from paired samples (tumor vs. healthy) revealed a robust over-expression of *INTS7, INTS8*, and *INTS13* genes in different tumors. As shown in Figure 5, the expression of these three genes is increased in cholangiocarcinoma, colon and lung adenocarcinomas, as well as in liver and lung squamous cell carcinomas. Additionally, *INTS7* is specifically over-expressed in breast cancer, whereas *INTS8* and *INTS13* is particularly over-expressed in kidney renal clear cell carcinoma.

**Figure 6:**
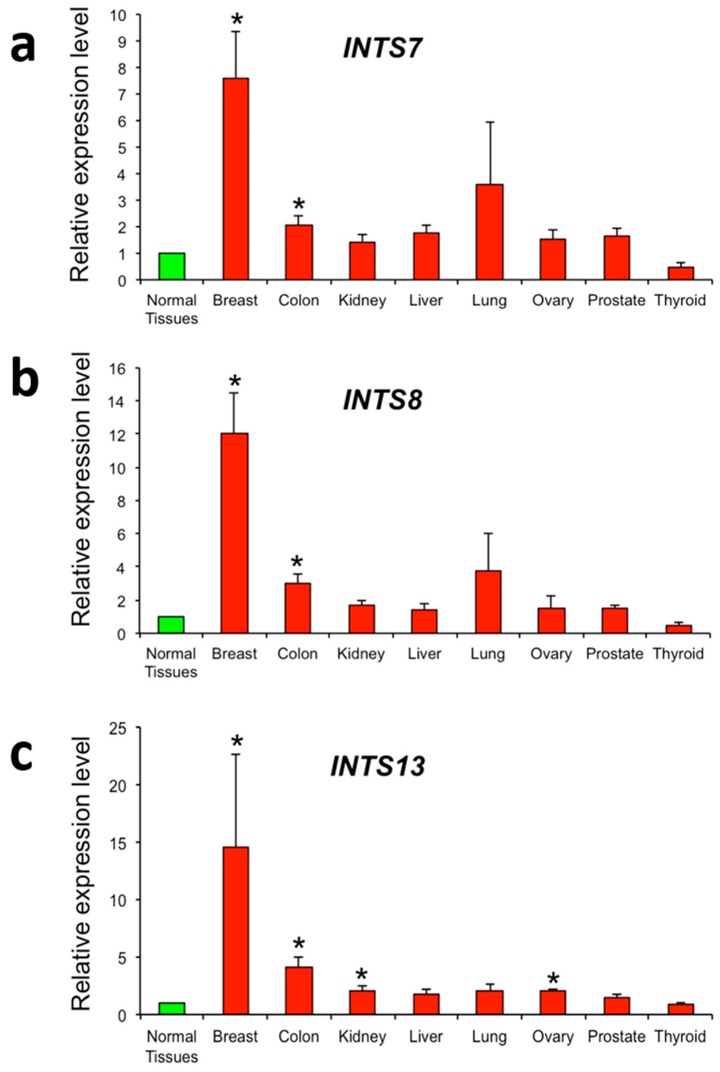
Relative expressions (mean ± ES) obtained by real-time PCR in breast, colon, kidney, liver, lung, ovary, prostate, and thyroid cancer tissues vs. corresponding normal tissues (with arbitrary expression value equal to 1). *INTS7* (**a**); *INTS8* (**b**) and *INTS13* (**c**). Significance: * *p* < 0.05 vs. normal tissues.

To validate these findings obtained from the analysis of TCGA datasets, we assayed a cDNA panel array containing eight different tumors (breast, colon, kidney, liver, lung, ovary, prostate, and thyroid). The panel contained normal and cancer tissues from independent patients diagnosed at various clinical disease stages and selected from mixed ages and genders. As illustrated in Figure 6, breast, colon, kidney, liver, lung, ovary, and prostate cancer tissues revealed a general over-expression of all analyzed genes. However, statistically significant differences in the expression of *INTS7, INTS8*, and *INTS13* between tumor and healthy samples were only measured for breast and colon cancers. Additionally, the *INTS13* gene was confirmed as being significantly overexpressed in kidney and ovarian tumors. Although not significant, a mild reduction of *INTS7*, *INTS8*, and *INTS13* expression was measured in thyroid cancer samples compared to normal ones.

## 3. Discussion

This is the first study providing a systematic and comprehensive overview of both the mutational status and the expression profile of all the genes encoding INT complex subunits across a large number of different cancer types. This complex plays key roles in transcription regulation and nucleic acid metabolism; besides, previous literature data indicated that some of the INT components are also be involved in human diseases, including many malignancies [2].

In the last few decades, the recent progress in high-throughput sequencing technology has contributed to the construction of genome-wide somatic mutation and transcription profiles in diverse cancer samples. In this regard, the large collection of multi-omic datasets available at the TCGA web portal represents a unique data source to study human cancers, especially for pan-cancer analysis. Particularly, genome-wide somatic alterations have been automatically catalogued starting from exome and whole-genome sequencing data in thousands of tumor samples. Similarly, gene expression—both at gene and transcript level—has been measured from RNA-Seq datasets in paired and unpaired tumor samples for the same cancer types [31].

The acquisition of somatic mutations is a key mechanism for the onset and progression of cancer, as well as for the sensitivity to chemotherapy. Thus, many researchers have tried to identify mutations causative of specific types of tumors, as well as to obtain a complete catalog of significantly mutated genes across all major cancer types [33,34]. Given the considerable number of somatic gene mutations found in tumor tissues, in the last few years, a huge effort has been employed in discerning mutated genes conferring a selective growth advantage (drivers) from those without a proven role in cancer and which have simply gradually accumulated randomly over the course of development or during uncontrolled cell growth (passengers) [33,35,36]. To date, several sophisticated mathematical tools have been developed to distinguish driver from passenger genes and to rank protein-coding genes using different strategies, such as the rate of cancer mutations over the background, the clustering patterns of mutations, and their functional impact [37]. Moreover, since many genes with an altered expression in tumor tissues can also provide a small growth advantage to tumor cells, a sub-classification was proposed to differentiate “Mut-driver genes”, usually altered by somatic gene mutations from “Epi-driver genes”, which are aberrantly expressed in tumors through epigenetic modifications, but are not frequently mutated [33]. In this regard, our pan-cancer investigation of mutations in the INT complex by OncodriveFML revealed that *INTS7* and *INTS8* can potentially be Mut-driver genes in endometrial carcinoma. However, considering that the mean number of somatic mutations in UCEC patients was about 850, an unusually high number of somatic mutations (from 5000 to 15,000) were identified in patients carrying mutations in *INTS7* and *INTS8*. Hypermutation is a frequent event in cancer, and recent whole-exome sequencing analyses have revealed that the “ultramutated” phenotype associates with somatic mutations in *POLE1*, even in endometrial carcinoma [38].

It is worth noting that the patients harboring mutations in *INTS7* and *INTS8* also have somatic mutations in *POLE1*. Endometrial cancers fall into four categories: *POLE* ultramutated, microsatellite instability hypermutated, copy number low, and copy number high [38]. According to TCGA classification, we cannot exclude the patients carrying *INTS7* and *INTS8* mutations from the *POLE* ultramutated subgroup. However, these patients are characterized by an increased C→A transversion frequency and improved progression-free survival. Noteworthy, in a very recent computational study, a multiscale mutation clustering algorithm was applied to identify variable length pan-cancer mutation clusters in cancer genes starting from an initial list of genes containing the highest ranked genes from MutSig; this analysis found multiscale clusters in 393 genes including, among others, *INTS7* [39].

Although our findings cannot definitely establish that these two genes are cancer drivers, it would be interesting to deepen these data in further analyses. The concept of a “driver” gene is gradually evolving and accordingly, new algorithms are being developed. Indeed, a recent comparative analysis of the currently available driver gene prediction methods has evaluated their performance, pointing out the strengths and weaknesses of each computational strategy and the lack of a gold standard [40]. Moreover, recent studies have highlighted the existence of genes, termed “mini-drivers”, with relatively weak tumor-promoting effects [41]. Multiple mutations in mini-driver genes might substitute for a major change in a known driver gene, especially in the presence of genomic instability or high mutagen exposure. Such a view is in line with a polygenic model of tumorigenesis. Another category has been also proposed: the so-called “latent drivers”, whose mutations behave as passengers. Usually, mutations in these genes do not confer a cancer phenotype, but when they occur with other mutations, they can drive cancer development and drug resistance [42]. The presence of “mini” and “latent” driver genes may be added to the list of explanations (technical issues, statistical power, exclusion of the non-coding regions, etc.) that account for the smaller than expected number of driver mutations observed in solid tumors. In this scenario, we cannot exclude the INTS genes from these alternative categories of driver genes. The development of new tailored computational methods will be used to definitely include (or exclude) the mutations in these genes from the growing list of cancer-driving events. It should be noted that known cancer driver genes are mainly involved in three core cellular processes: cell fate, cell survival, and genome maintenance. Interestingly, the Integrator complex has a relevant role in all of them. Specifically, IntS7 participates with other INT subunits and NABPs in the formation of protein complexes involved in the DNA damage response and in genome maintenance [2,13,14,15,16,43]. Indeed, its siRNA-mediated depletion determines cell cycle arrest bypass and mitomycin C sensitivity [2,43]. Additionally, pan-cancer studies are gradually highlighting that individual mutations (even at a low frequency) tend to converge—in a particular type of cancer—into specific cellular pathways, rather than into specific genes. This hypothesis has encouraged the development of novel computational approaches for evaluating cancer somatic mutation data that are based on pathways or protein complexes network analyses [44,45], rather than on the sole mutation frequency of a specific gene.

Remarkably, despite not being able to definitely establish *INTS7* and *INTS8* as cancer driver genes, the transcriptome analysis of RNA-Seq paired samples revealed that these two genes, together with *INTS13*, are the most deregulated across cancers. These data suggest that they may act as Epi-driver genes, rather than as Mut-drivers. Of note, we experimentally validated their transcriptional alteration in primary breast and colon tumor samples (Figure 6), confirming TCGA data re-analysis. Interestingly, microarray- and exome sequencing-based studies proposed a role of *INTS8* in gastric cancer and peripheral T-cell lymphoma [26,27]. Unfortunately, freely available data from TCGA (tier 1) did not include RNA-Seq data from paired stomach adenocarcinoma (STAD) samples and datasets of peripheral T-cell lymphoma (see Table 1).

Interestingly, we found that *INTS6*, also known as *DICE1* (deleted in cancer 1), was downregulated in several cancer types, thus corroborating previous literature data and supporting its role as a tumor suppressor gene [17,18,19,20,21,22,23].

Our results can be useful to restrict the attention to a subset of relevant INT subunits deserving further targeted investigations. Indeed, some of the genes described in this work as frequently mutated and/or transcriptionally deregulated might have a potential impact on tumor initiation and progression. We are aware that functional studies on specific INT gene mutations should be performed to definitely prove their potential oncogenic role. It would also be desirable to investigate whether these mutations contribute to cancer progression and survival, other than their relation with drug-response and -resistance, through follow-up studies examining the mutational status of INT subunits in lymph node and distant metastases tissues. Moreover, molecular studies addressing the (epi)genetic changes underlying the altered gene expression observed in tumor samples are needed. Finally, the effect of protein interactions between INT subunits and other partners involved in the genome maintenance pathways needs to be investigated. Indeed, recent studies revealed that other subunits (such as NABPs) belong to this complex [2], and it would be relevant to investigate whether mutations in the genes encoding these proteins or their deregulation can also contribute to cancer.

The availability of data produced from TCGA and other large consortia is offering the unique opportunity to easily access large catalogues of single omic datasets to investigate distinct molecular aspects of many cancer types. However, despite having provided the possibility to formulate new biological hypotheses (that need experimental validation), it has posed new challenges. Indeed, the impact of such data cannot be fully exploited without the development of multi-omic data integration tools and methods [46]. In this regard, we envisage that our analysis of INT gene expression could be further integrated by a systematic pan-cancer study of the epigenetic marks in these genes.

## 4. Materials and Methods

### 4.1. TCGA Data Source Selection and Processing for Mutation Analysis

All the data used in this work (exome- and RNA-Seq data) were downloaded from The Cancer Genome Atlas, [47]. In order to analyze the Exome-Seq data, all files containing somatic mutations in a .maf (Mutation Annotation File) format were downloaded for every human primary cancer. Since such data were analyzed from several consortia, all files retrieved for each cancer type were merged. The number of samples for each cancer type is illustrated in Table 1.

The selection and nomenclature of INT genes were based on the HUGO Gene Nomenclature Committee [48]. Analyses of the mutational landscape of INT subunit genes (i.e., evaluation of mutated patients for each subunit, Mann–Whitney test, and identification of frequently mutated sites) were performed building a customized computational pipeline in R programming language. Only non-synonymous mutations were considered for further analyses.

To estimate the positive selection and the accumulated functional impact (FI) bias for the somatic mutations falling within the coding region of INT genes, we used OncodriveFML [32,49]. OncodriveFML was run using default parameters and the statistical significance values were set as reported in Mularoni et al., 2016. OncodriveFML used precomputed Combined Annotation-Dependent Depletion (CADD) scores for functional impact bias (obtained via OncodriveFML) and a file reporting the genomic coordinates of the coding sequences (CDS) from the OncodriveFML website 

### 4.2. TCGA Data Source Selection and Processing for Expression Analysis

The analysis of gene expression and the identification of differentially expressed genes were performed comparing the expression profiles of cancer vs. normal samples within the same patient in a paired analysis. Therefore, expression data taken from human primary cancers for which healthy samples were not available were discarded. According to this criterion, 22 tumor entities were analyzed. In order to have a more robust differential expression analysis in paired samples, we applied generalized linear models (GLM) implemented in the EdgeR Bioconductor package version 3.17.10. Multiple correction was performed through the application of the false discovery rate (FDR) method. We considered differentially expressed genes with a logFC ≤ −1 and logFC ≥ 1, and an FDR ≤ 0.01.

### 4.3. Real-Time RT-PCR Analysis

Quantitative Real-Time PCR (qRT-PCR) experiments were carried out on TissueScan Cancer Survey Panels (OriGene, Rockville, MD). TissueScan Cancer Survey Panels were purchased in a 96-well format with lyophilized cDNA samples from various normal and tumor tissues covering eight different cancers (breast, colon, kidney, liver, lung, ovary, prostate, and thyroid).

To quantitatively determine the relative amount of *INTS7*, *INTS8*, and *INTS13* RNAs, qRT-PCR was performed using a Bio-Rad iQ iCycler Detection System (Bio-Rad Laboratories, Ltd. Hercules, California 94547, USA) with SYBR green fluorophore. The amplification reaction mix contained 2X SSoAdvanced Universal SYBR Green Supermix (Bio-Rad Laboratories), 10 pmol/uL of each primer. The conditions used were: First denaturation at 95 °C for 2 min, followed by 40 cycles of 95 °C for 5 s and 60 °C for 30 s. Primers were designed using Primer3Plus [50]. The specificity of each oligonucleotide pair used was verified with the BLAST program and through in-silico PCR analysis by UCSC-Genome Browser [51].

The selected sequences of oligonucleotides were: *INTS7* forward 5′-AAG TCA AAA CCG AAG AAA TGC-3′; *INTS7* reverse 5′-CCC TGG CAT TTT CAT AGA CA-3′; *INTS8* forward 5′-AAG TCA AAA CCG AAG AAA TGC-3′; *INTS8* reverse 5′-CCC TGG CAT TTT CAT AGA CA-3′; *INST13* forward 5′-GAC AAG TCA GAG AAA GCA GT-3′; and *INST13* reverse 5′-GGG GAA TCA GGC GAA TCT TT-3′.

The amplification conditions for each primer pair were experimentally determined. The amplification products were also analyzed by agarose gel electrophoresis [52]. Data were normalized with β-actin (*ACTB* gene) that was provided with TissueScan Cancer Survey Panels. Melting curves were generated after amplification; the relative gene expression was calculated using the 2^−ΔΔ*C*t^ method [53]. The results are expressed as the mean ± ES. The statistical significance of differences between experimental groups was calculated using the unpaired two-tailed Student’s *t*-test. Results with a *p*-value < 0.05 were considered significant.

## Figures and Tables

**Table 1:** List of cancer types and number of patients (*n*) analyzed from TCGA (The Cancer Genome Atlas).

Abbreviation	Cancer Type	Mutation Analysis *n*	Expression Analysis *n* (Paired)
ACC	Adrenocortical carcinoma	92	-
BLCA	Bladder cancer	412	19
BRCA	Breast cancer	1098	57
CESC	Cervical squamous cell carcinoma and endocervical adenocarcinoma	308	3
CHOL	Cholangiocarcinoma	51	9
COAD	Colon adenocarcinoma	463	26
DLBC	Lymphoid neoplasm diffuse large B-cell lymphoma	58	-
ESCA	Esophageal carcinoma	185	13
GBM	Glioblastoma	617	5
HNSC	Head and neck squamous cell carcinoma	528	43
KICH	Kidney chromophobe carcinoma	113	25
KIRC	Kidney renal clear cell carcinoma	537	72
KIRP	Kidney renal papillary cell carcinoma	291	32
LAML	Acute myeloid leukemia	200	-
LIHC	Liver hepatocarcinoma	377	50
LUAD	Lung adenocarcinoma	585	58
LUSC	Lung squamous cell carcinoma	504	51
OV	Ovarian cancer	608	-
PAAD	Pancreas adenocarcinoma	185	51
PCPG	Pheochromocytoma and paraganglioma	179	4
PRAD	Prostate adenocarcinoma	500	3
READ	Rectum adenocarcinoma	172	52
SARC	Sarcoma	261	2
SKCM	Skin cutaneous melanoma	470	-
STAD	Stomach adenocarcinoma	478	-
TGCT	Testicular germ cell tumors	150	-
THCA	Thyroid cancer	507	57
THYM	Thymoma	124	2
UCEC	Uterine corpus endometrial carcinoma	560	7
UCS	Uterine carcinosarcoma	57	-
UVM	Uveal melanoma	80	-

## Supplementary Materials

Supplementary materials can be found at www.mdpi.com/1422-0067/18/5/936/s1.

## Abbreviations

INT	Integrator complex
NABP	Nucleic acid binding proteins
TCGA	The Cancer Genome Atlas
FC	Fold change
FDR	False discovery rate
