# Supplementary material for: Pan-Cancer Mutational and Transcriptional Analysis of the Integrator Complex
Source: Int J Mol Sci. 2017 Apr 29;18(5):936. doi: 10.3390/ijms18050936 (PMC5454849; doi:10.3390/ijms18050936)
Supplement: Supplementary file 1 [file ijms-18-00936-s001.zip › Supplementary_files/Supplementary_file_S1.pdf]

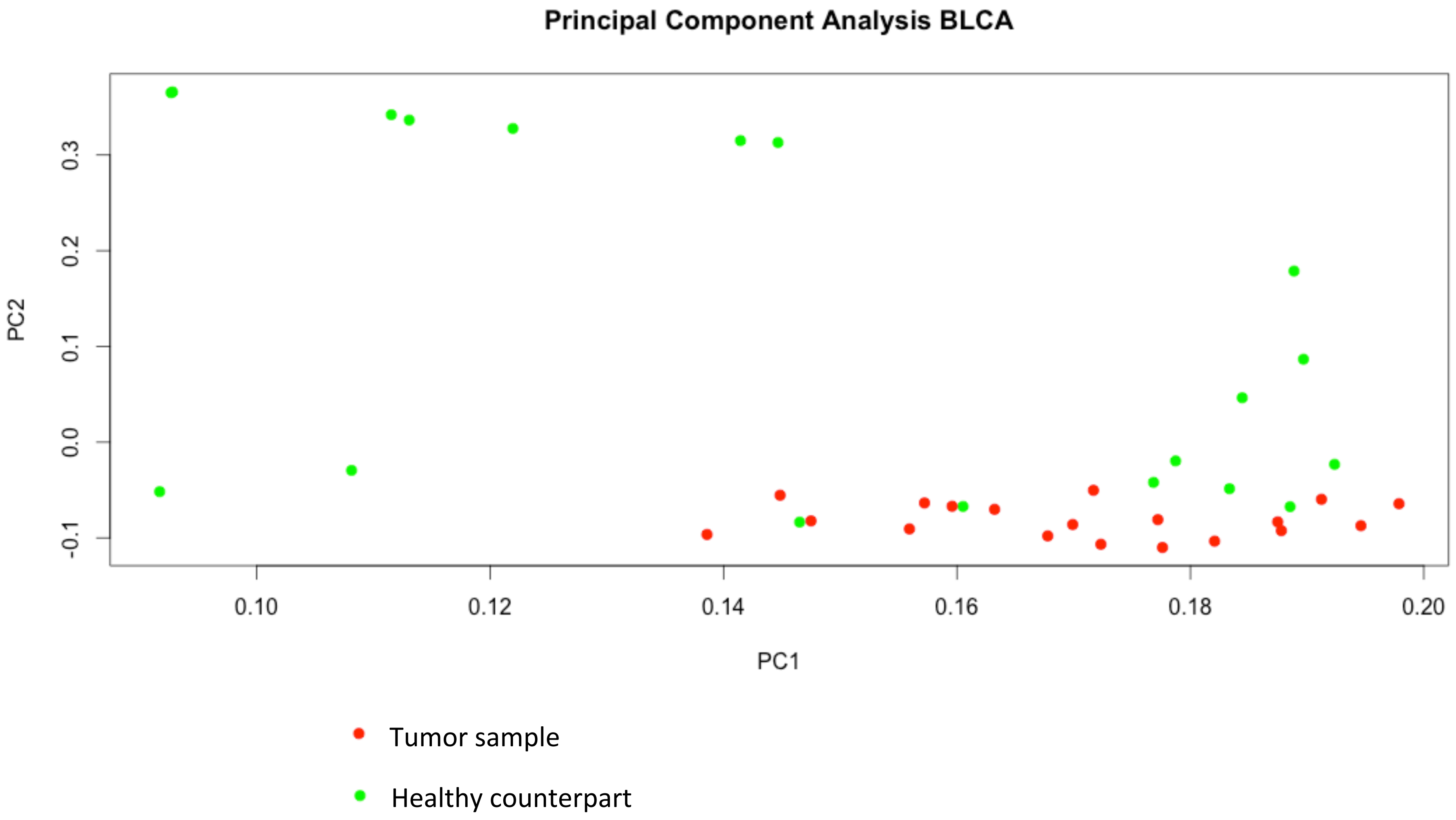

Principal Component Analysis BLCA
PC2
0.3
0.2
0.1
0.0
-0.1
0.10
0.12
0.14
0.16
0.18
0.20
PC1
Tumor sample
Healthy counterpart

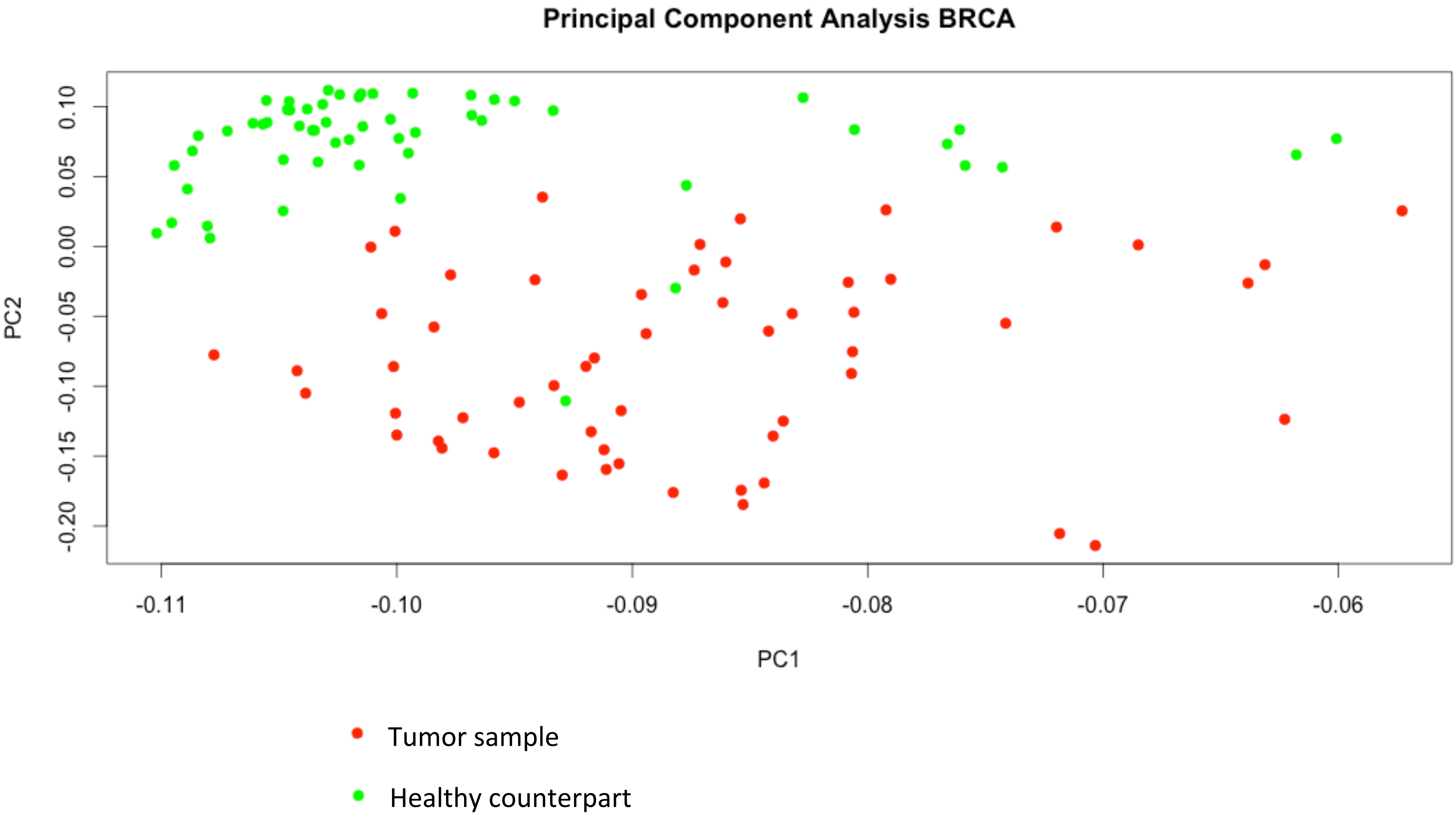
Principal Component Analysis BRCA
PC1
PC2
-0.11
-0.10
-0.09
-0.08
-0.07
-0.06
0.10
0.05
0.00
-0.05
-0.10
-0.15
-0.20
Tumor sample
Healthy counterpart

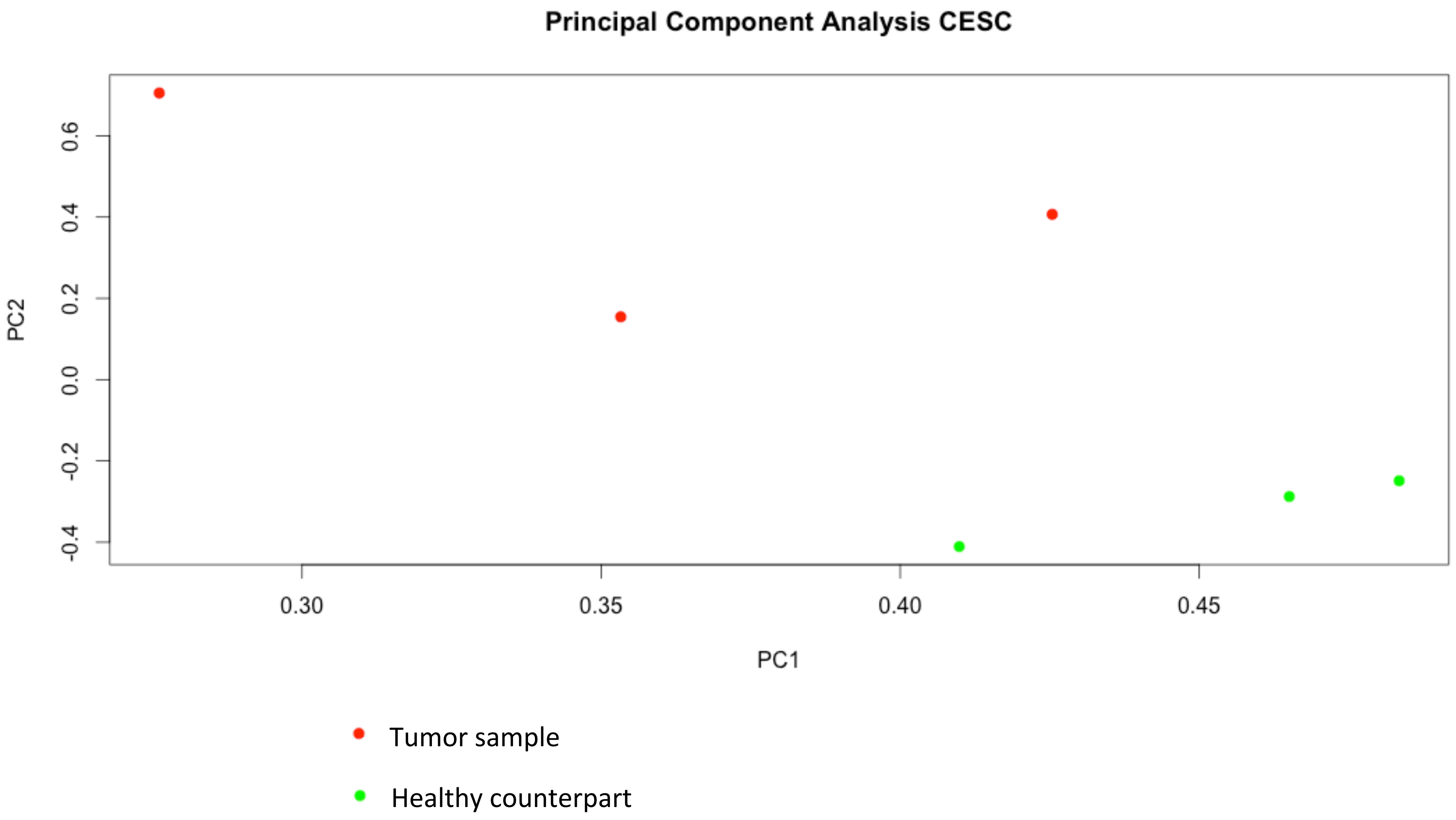
Principal Component Analysis CESC
PC2
0.6
0.4
0.2
0.0
-0.2
-0.4
0.30
0.35
0.40
0.45
PC1
Tumor sample
Healthy counterpart

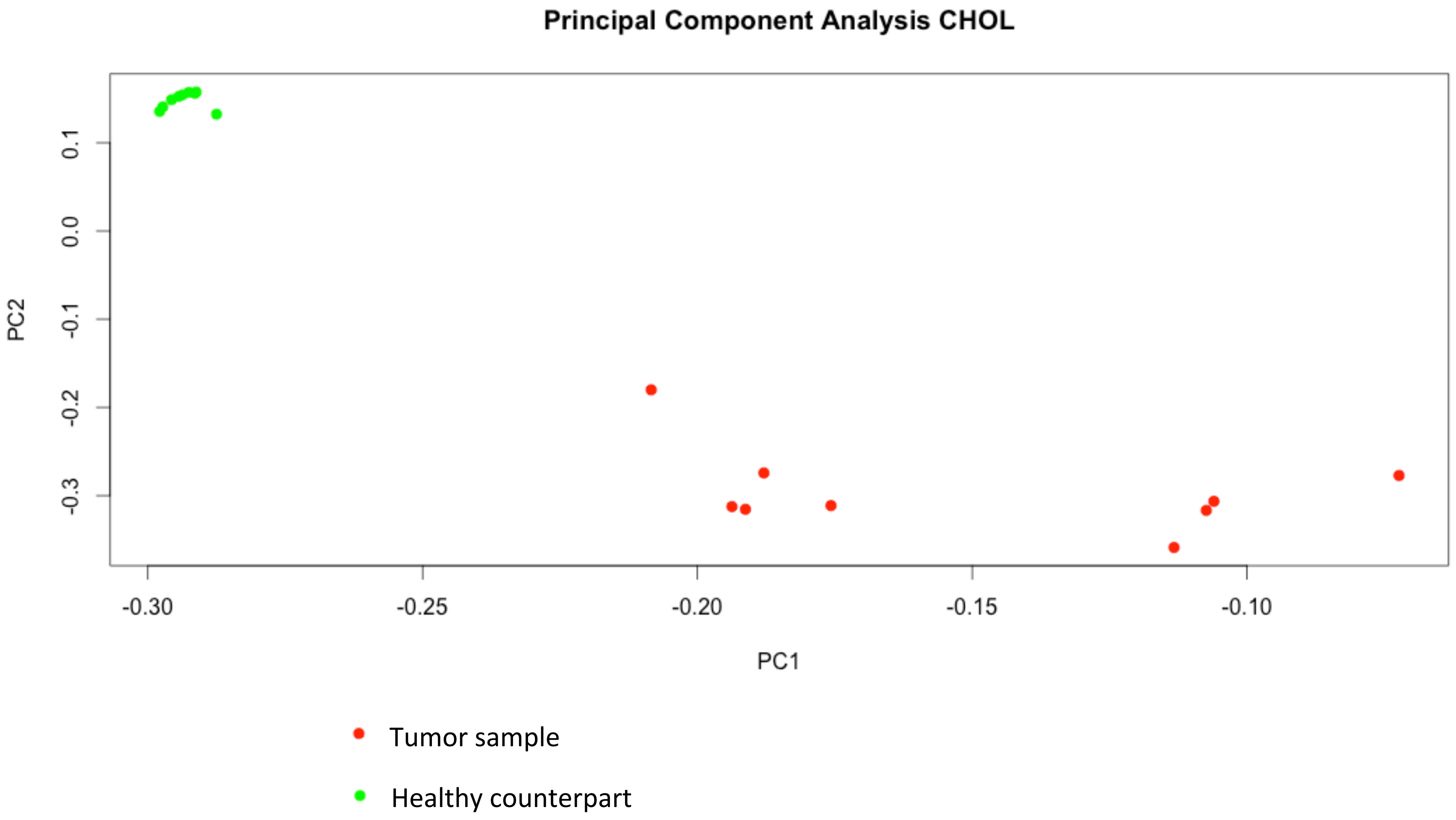
Principal Component Analysis CHOL
PC2
0.1
0.0
-0.1
-0.2
-0.3
-0.30
-0.25
-0.20
-0.15
-0.10
PC1
Tumor sample
Healthy counterpart

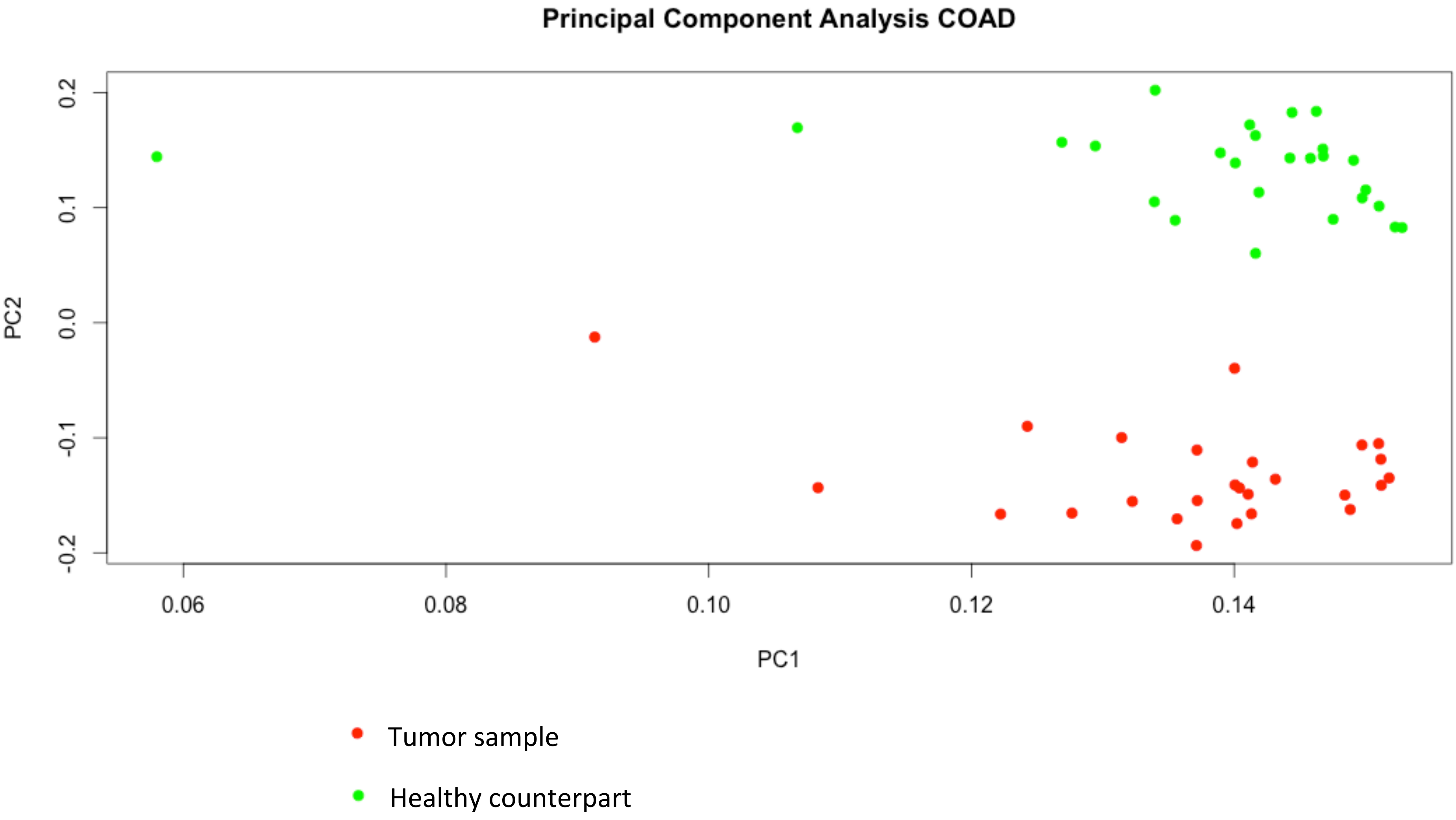
Principal Component Analysis COAD
PC2
0.2
0.1
0.0
-0.1
-0.2
0.06
0.08
0.10
0.12
0.14
PC1
Tumor sample
Healthy counterpart

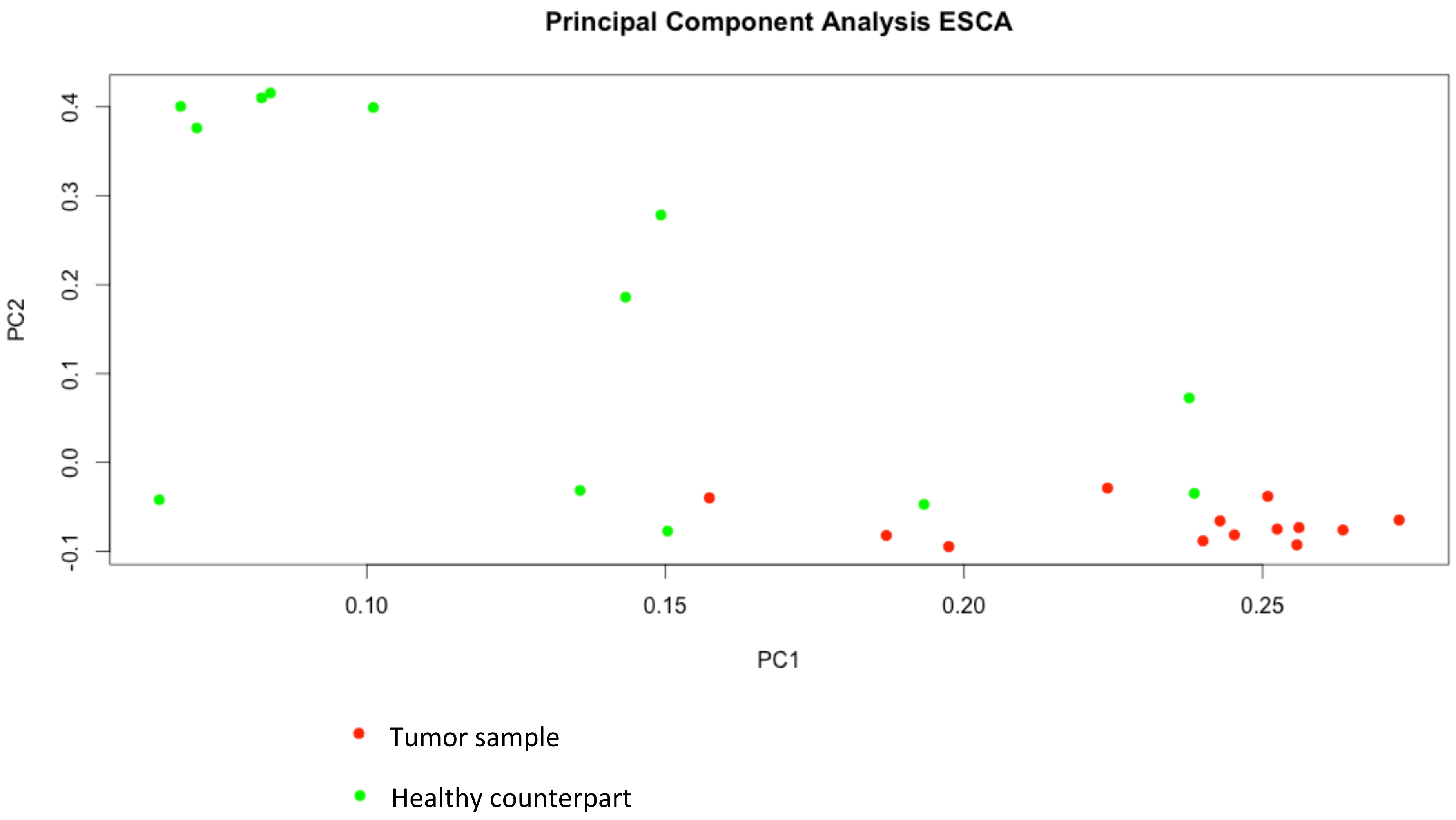
Principal Component Analysis ESCA
PC2
-0.1
0.0
0.1
0.2
0.3
0.4
0.10
0.15
0.20
0.25
PC1
Tumor sample
Healthy counterpart

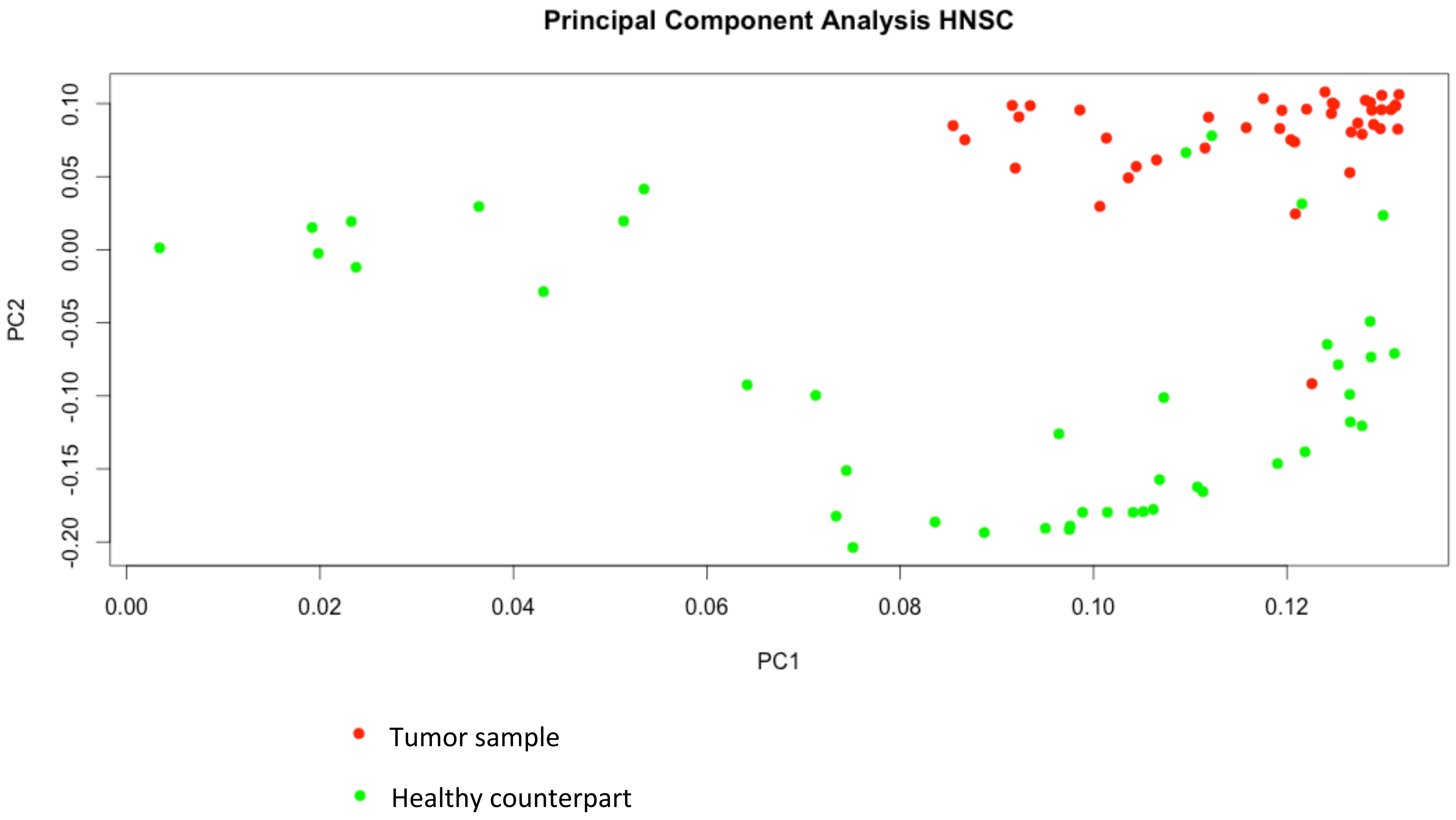
Principal Component Analysis HNSC
PC2
0.10
0.05
0.00
-0.05
-0.10
-0.15
-0.20
PC1
0.00
0.02
0.04
0.06
0.08
0.10
0.12
Tumor sample
Healthy counterpart

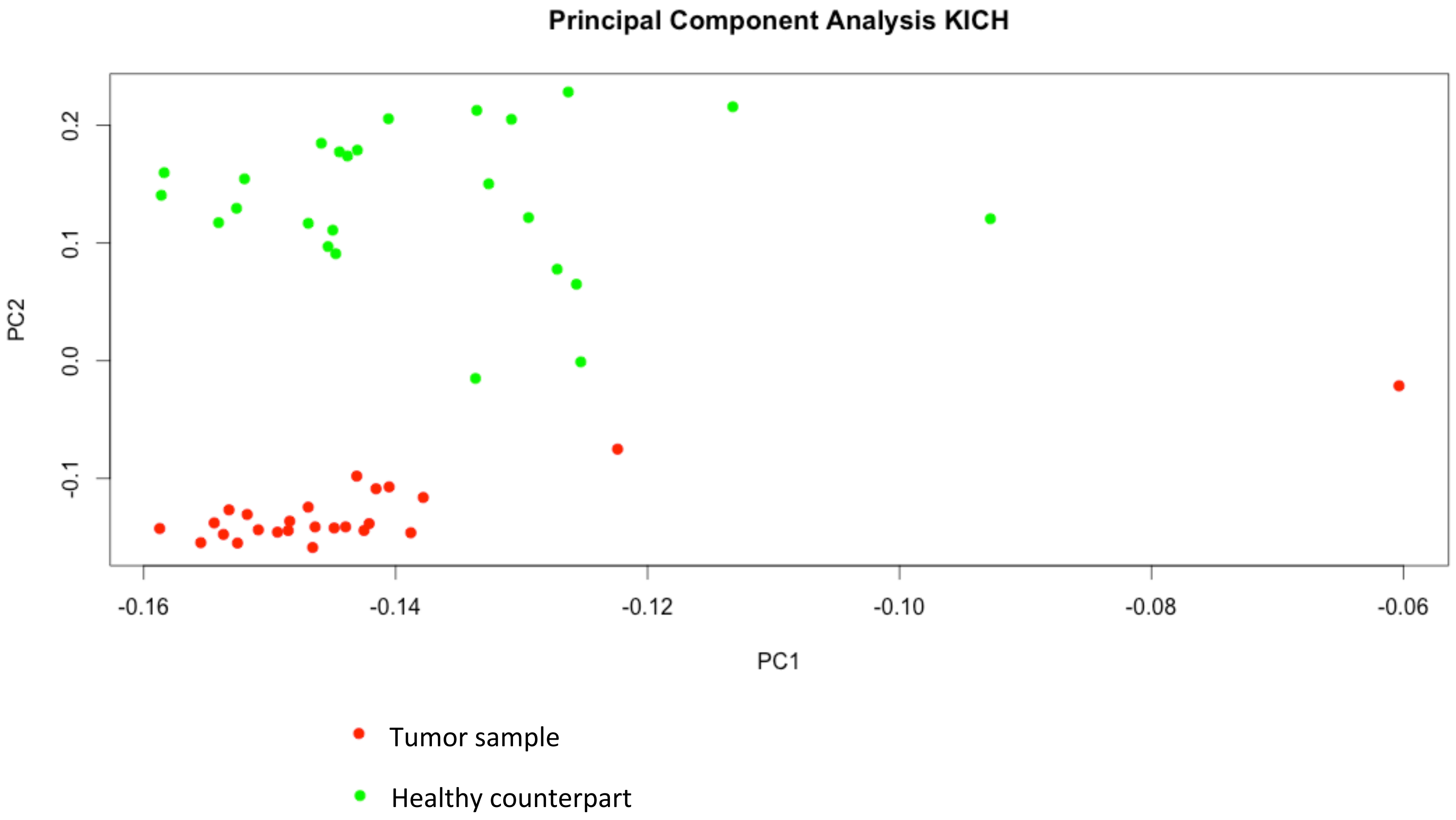
Principal Component Analysis KICH
PC2
0.2
0.1
0.0
-0.1
-0.16
-0.14
-0.12
-0.10
-0.08
-0.06
PC1
Tumor sample
Healthy counterpart

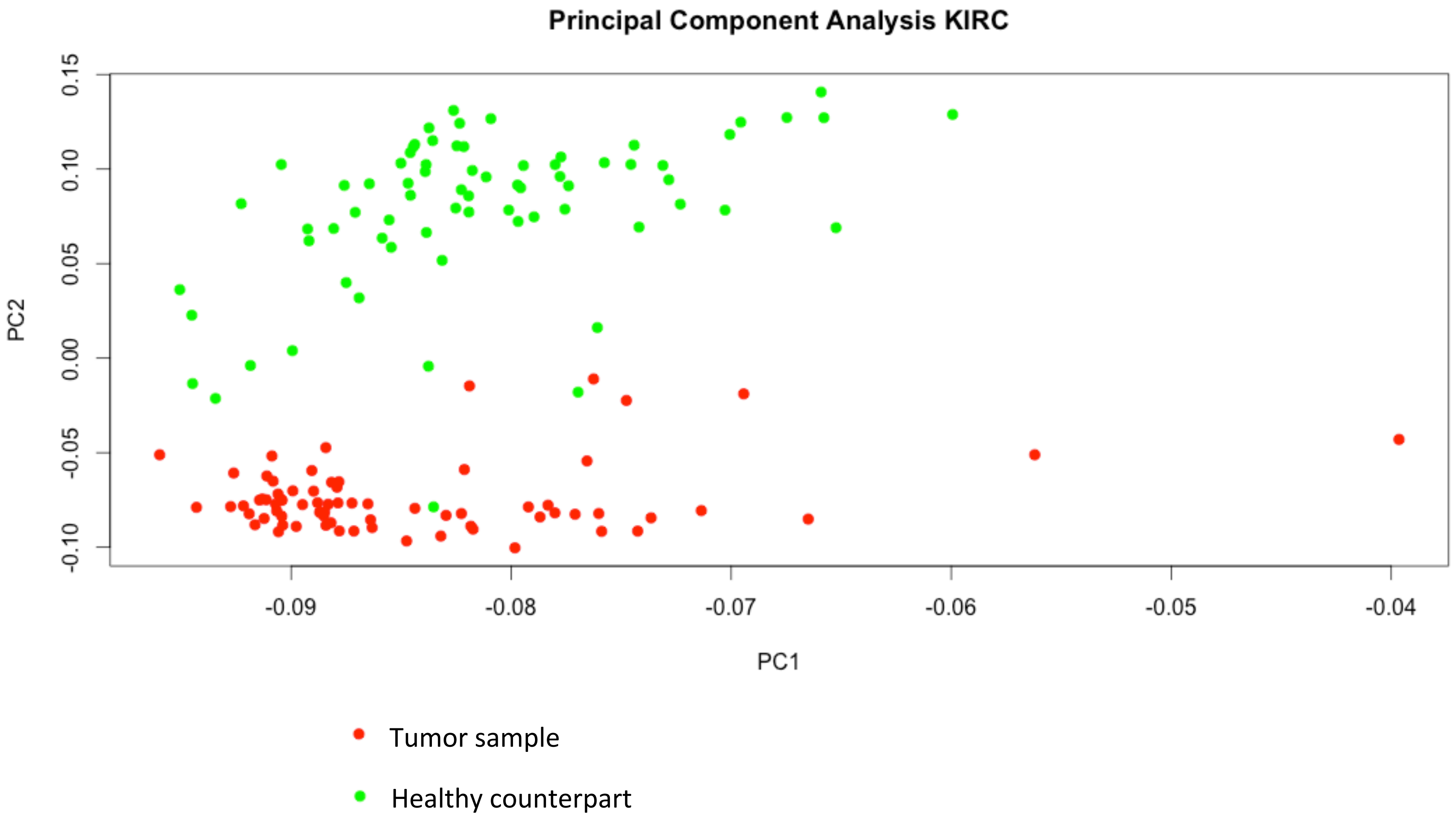
Principal Component Analysis KIRC
PC2
0.15
0.10
0.05
0.00
-0.05
-0.10
PC1
-0.09
-0.08
-0.07
-0.06
-0.05
-0.04
Tumor sample
Healthy counterpart

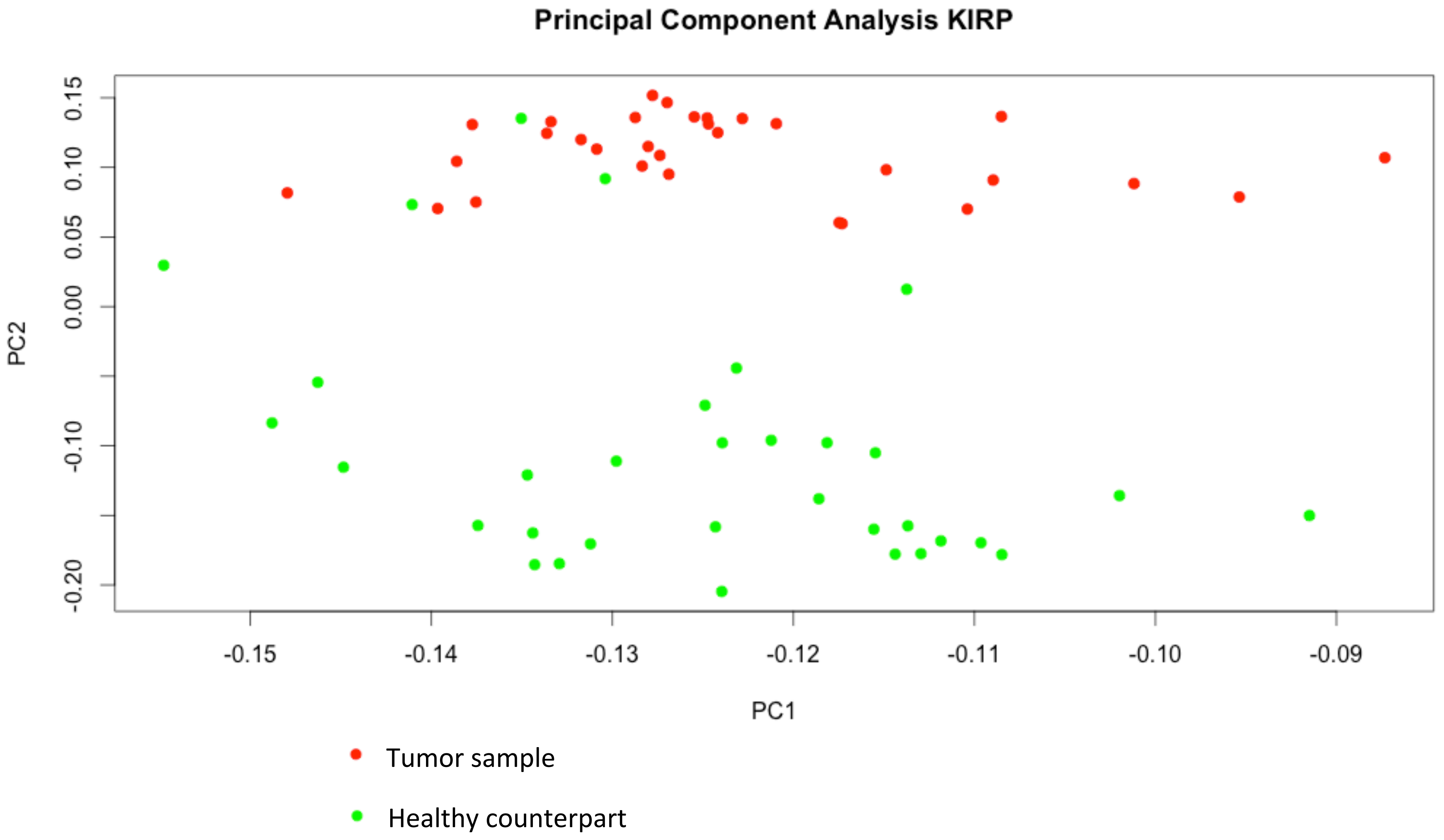
Principal Component Analysis KIRP
PC2
0.15
0.10
0.05
0.00
-0.10
-0.20
-0.15
-0.14
-0.13
-0.12
-0.11
-0.10
-0.09
PC1
Tumor sample
Healthy counterpart

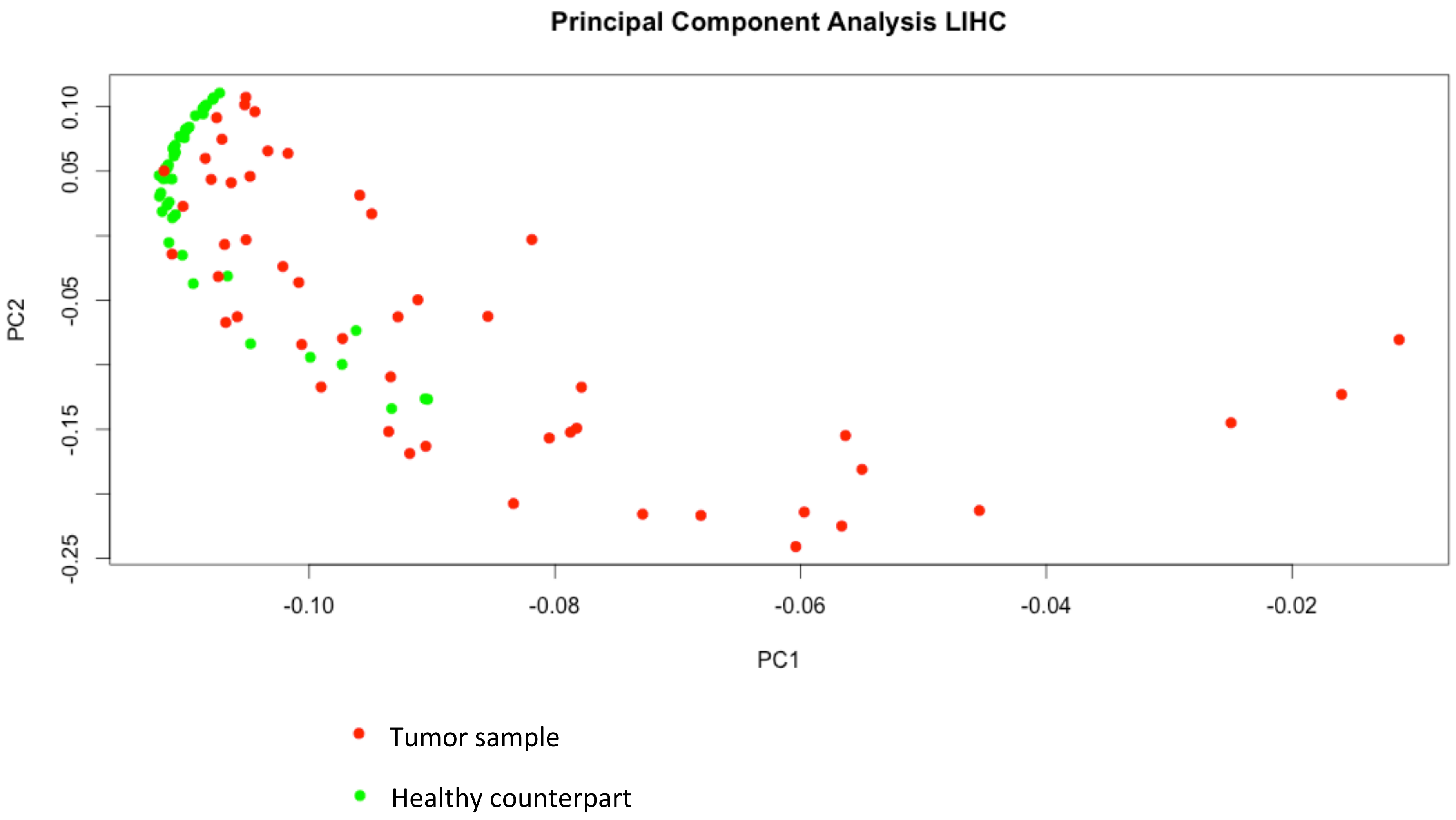
Principal Component Analysis LIHC
PC2
0.10
0.05
-0.05
-0.15
-0.25
-0.10
-0.08
-0.06
-0.04
-0.02
PC1
Tumor sample
Healthy counterpart

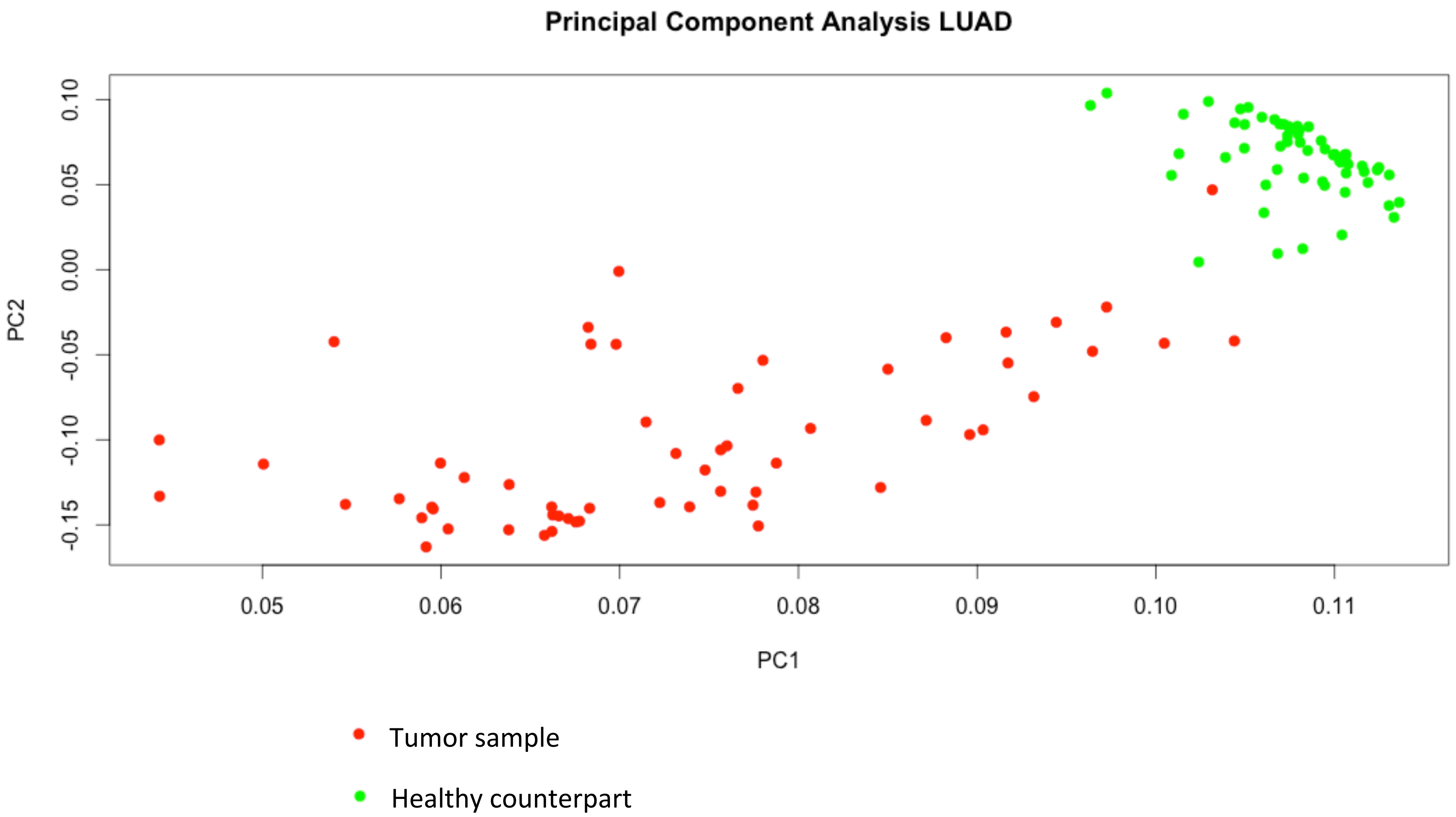

Principal Component Analysis LUAD
PC2
0.10
0.05
0.00
-0.05
-0.10
-0.15
0.05
0.06
0.07
0.08
0.09
0.10
0.11
PC1
Tumor sample
Healthy counterpart

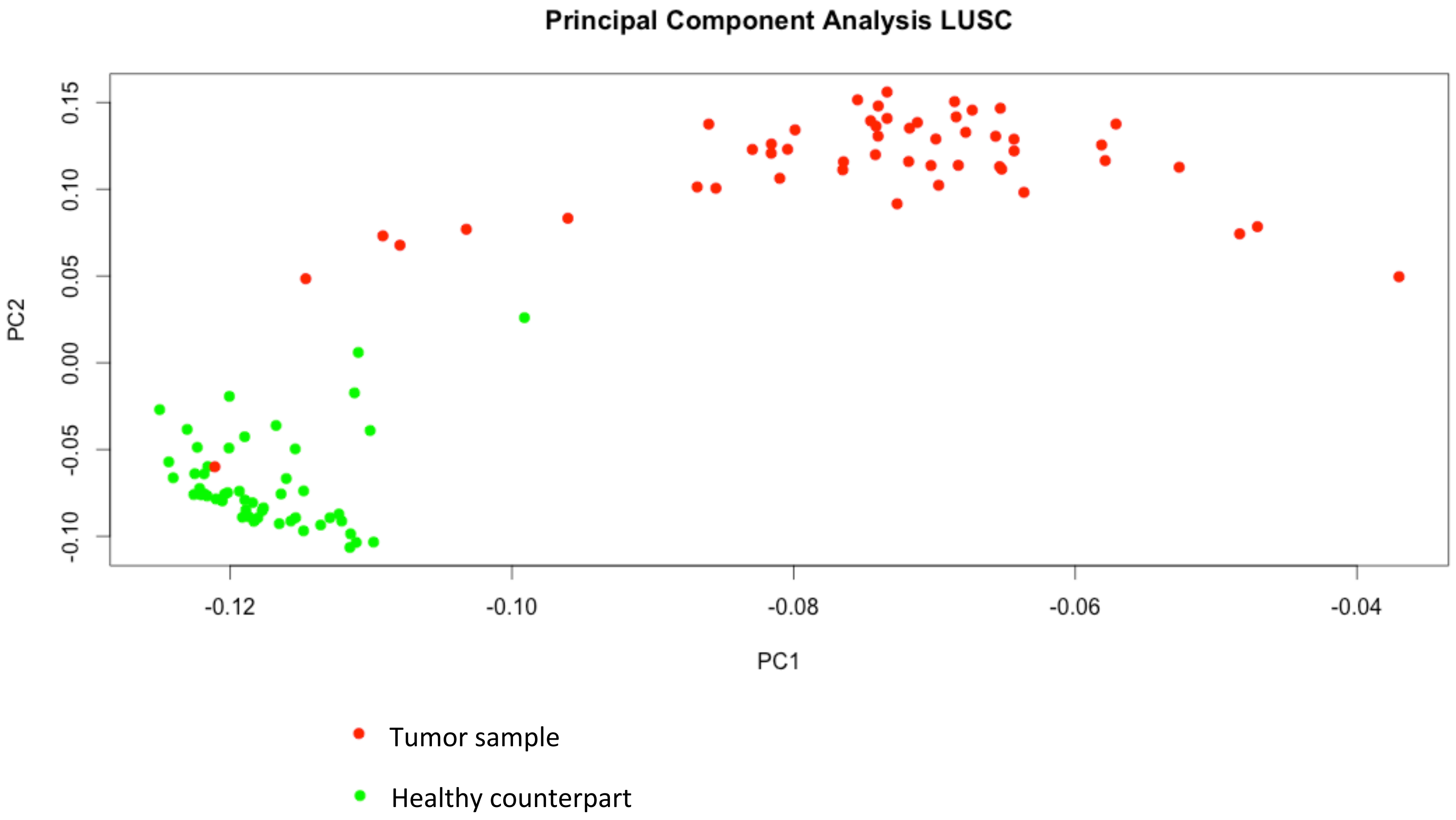

Principal Component Analysis LUSC
PC2
0.15
0.10
0.05
0.00
-0.05
-0.10
-0.12
-0.10
-0.08
-0.06
-0.04
PC1
Tumor sample
Healthy counterpart

Principal Component Analysis PAAD

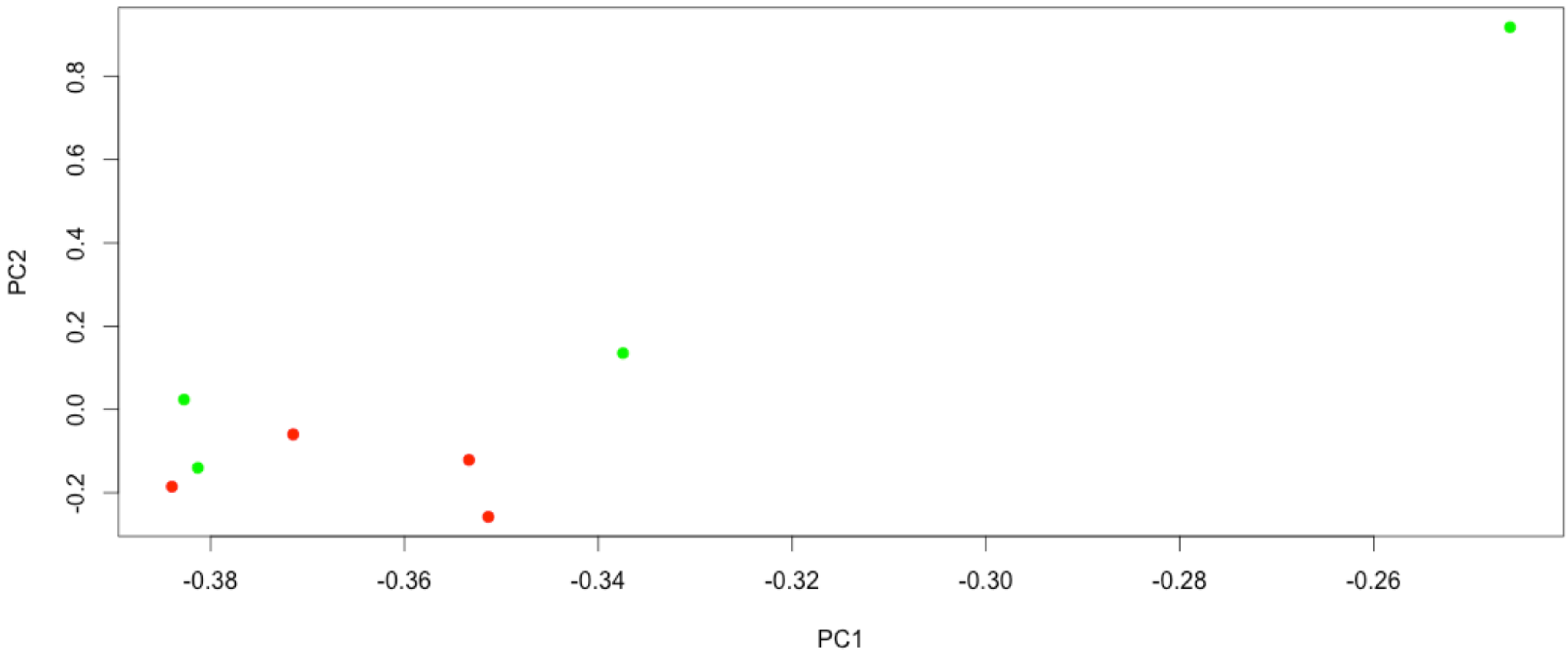

Tumor sample

Healthy counterpart

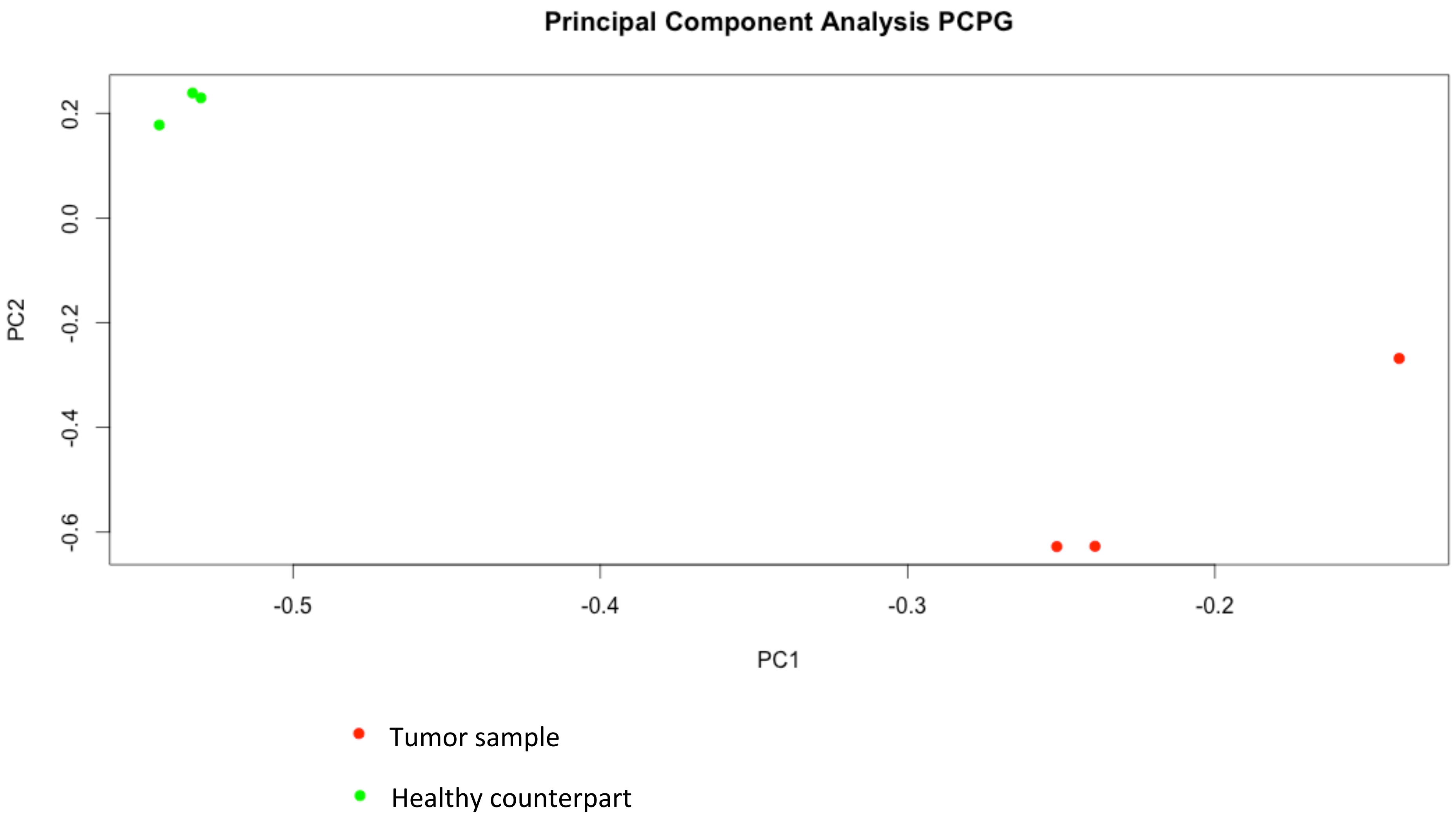
Principal Component Analysis PCPG
PC2
0.2
0.0
-0.2
-0.4
-0.6
-0.5
-0.4
-0.3
-0.2
PC1
Tumor sample
Healthy counterpart

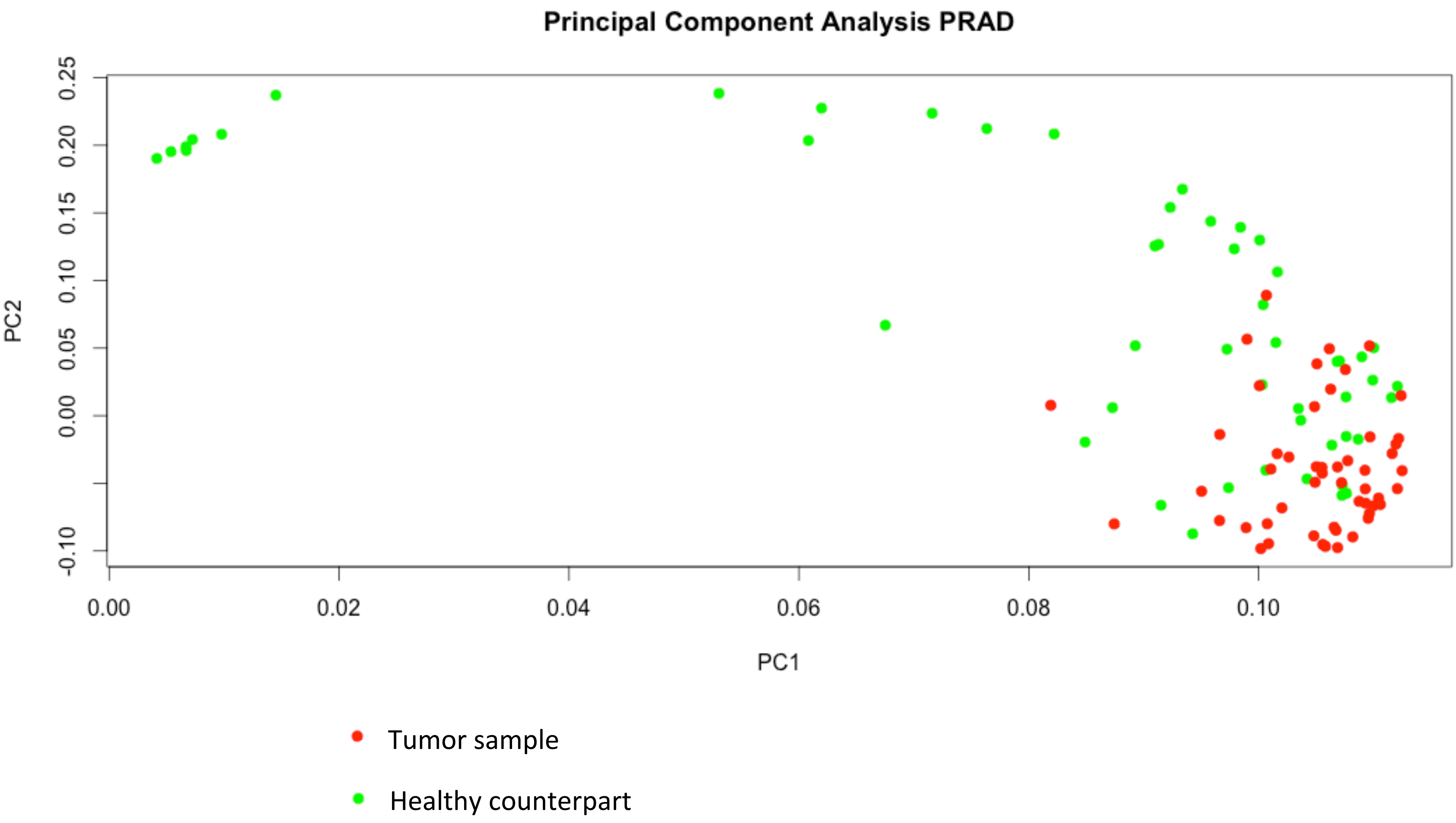
Principal Component Analysis PRAD
PC2
0.25
0.20
0.15
0.10
0.05
0.00
-0.10
0.00
0.02
0.04
0.06
0.08
0.10
PC1
Tumor sample
Healthy counterpart

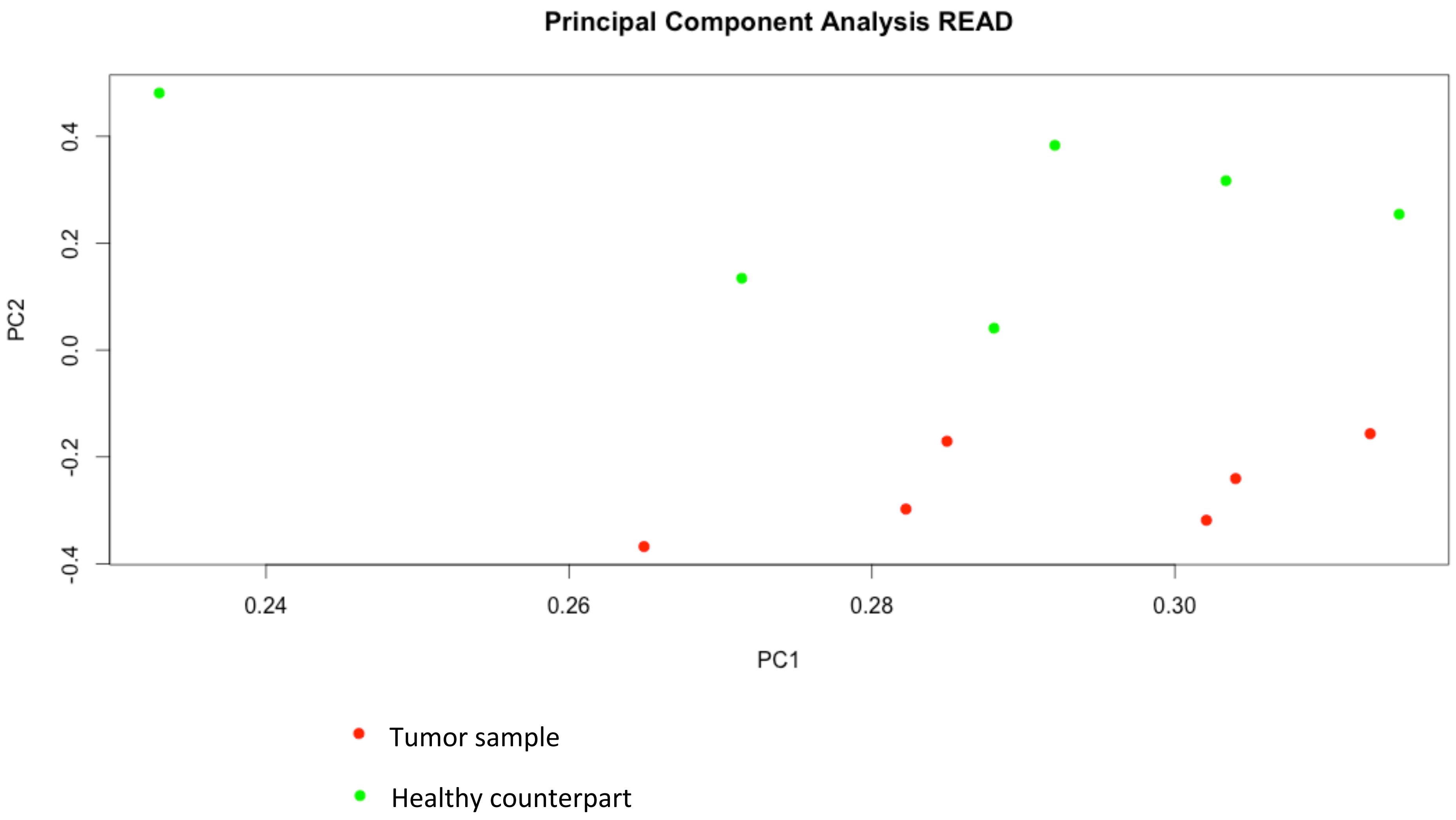
Principal Component Analysis READ
PC2
0.4
0.2
0.0
-0.2
-0.4
0.24
0.26
0.28
0.30
PC1
Tumor sample
Healthy counterpart

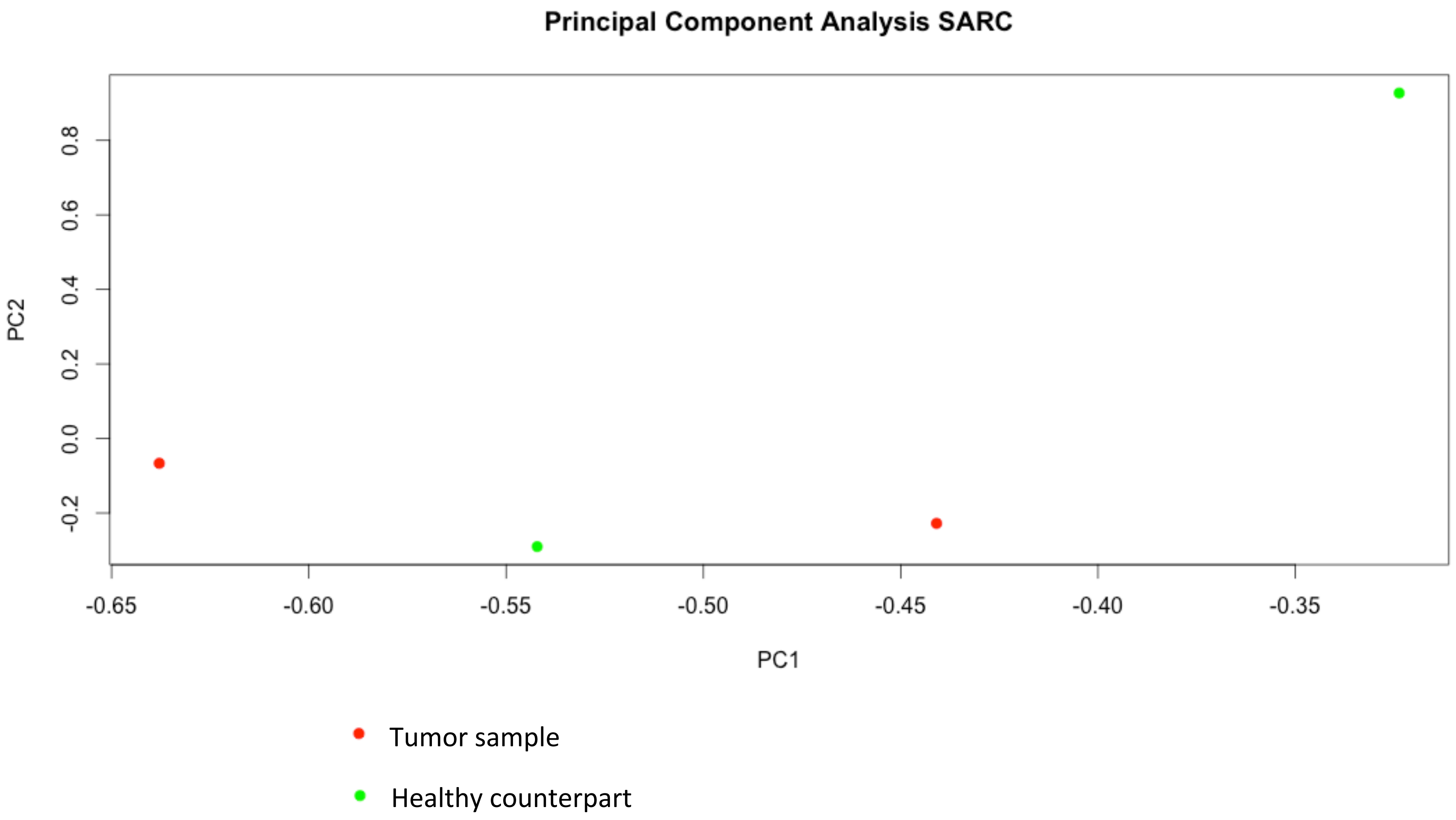
Principal Component Analysis SARC
PC2
0.8
0.6
0.4
0.2
0.0
-0.2
-0.65
-0.60
-0.55
-0.50
-0.45
-0.40
-0.35
PC1
Tumor sample
Healthy counterpart

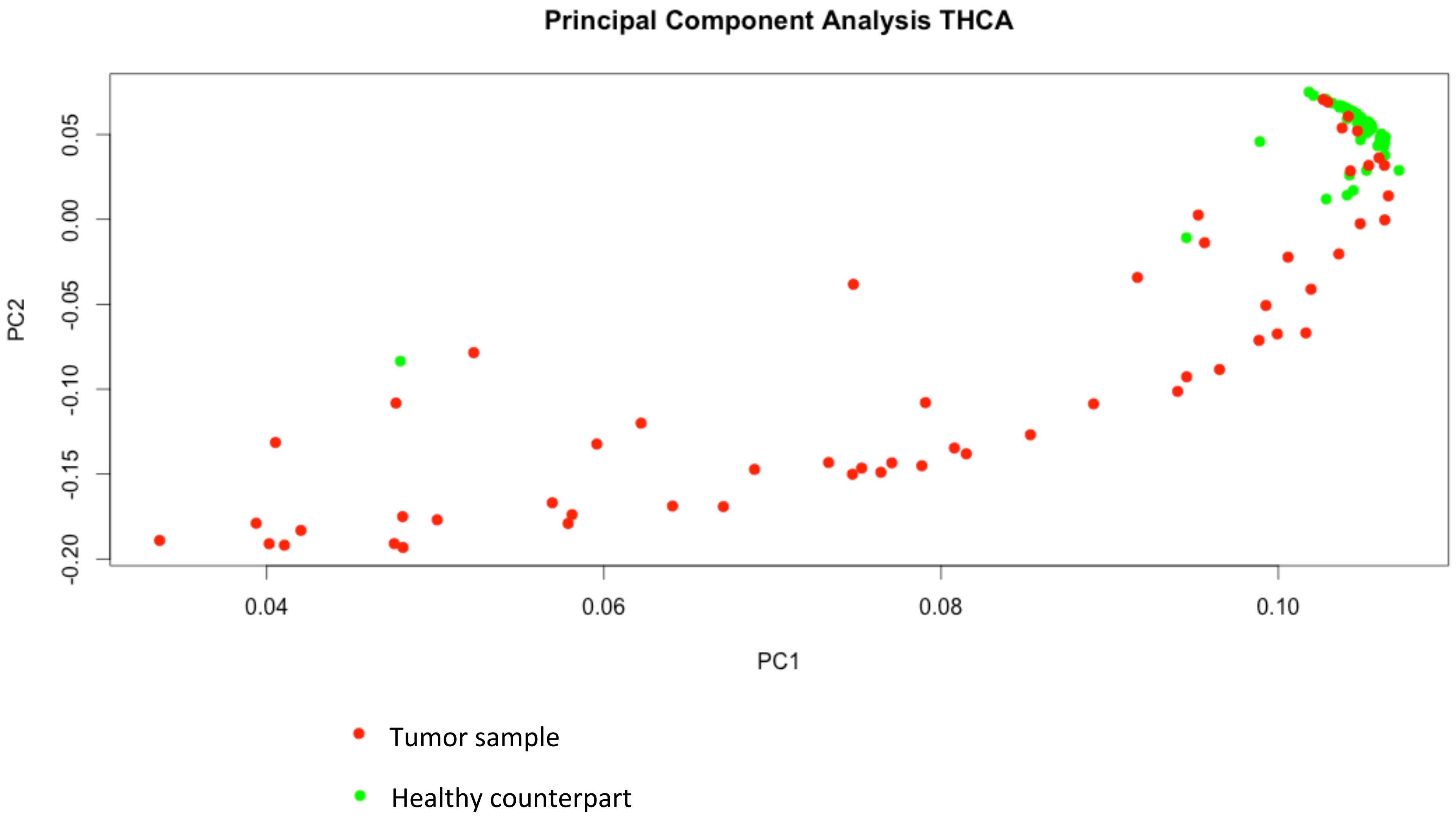
Principal Component Analysis THCA
PC2
0.05
0.00
-0.05
-0.10
-0.15
-0.20
0.04
0.06
0.08
0.10
PC1
Tumor sample
Healthy counterpart

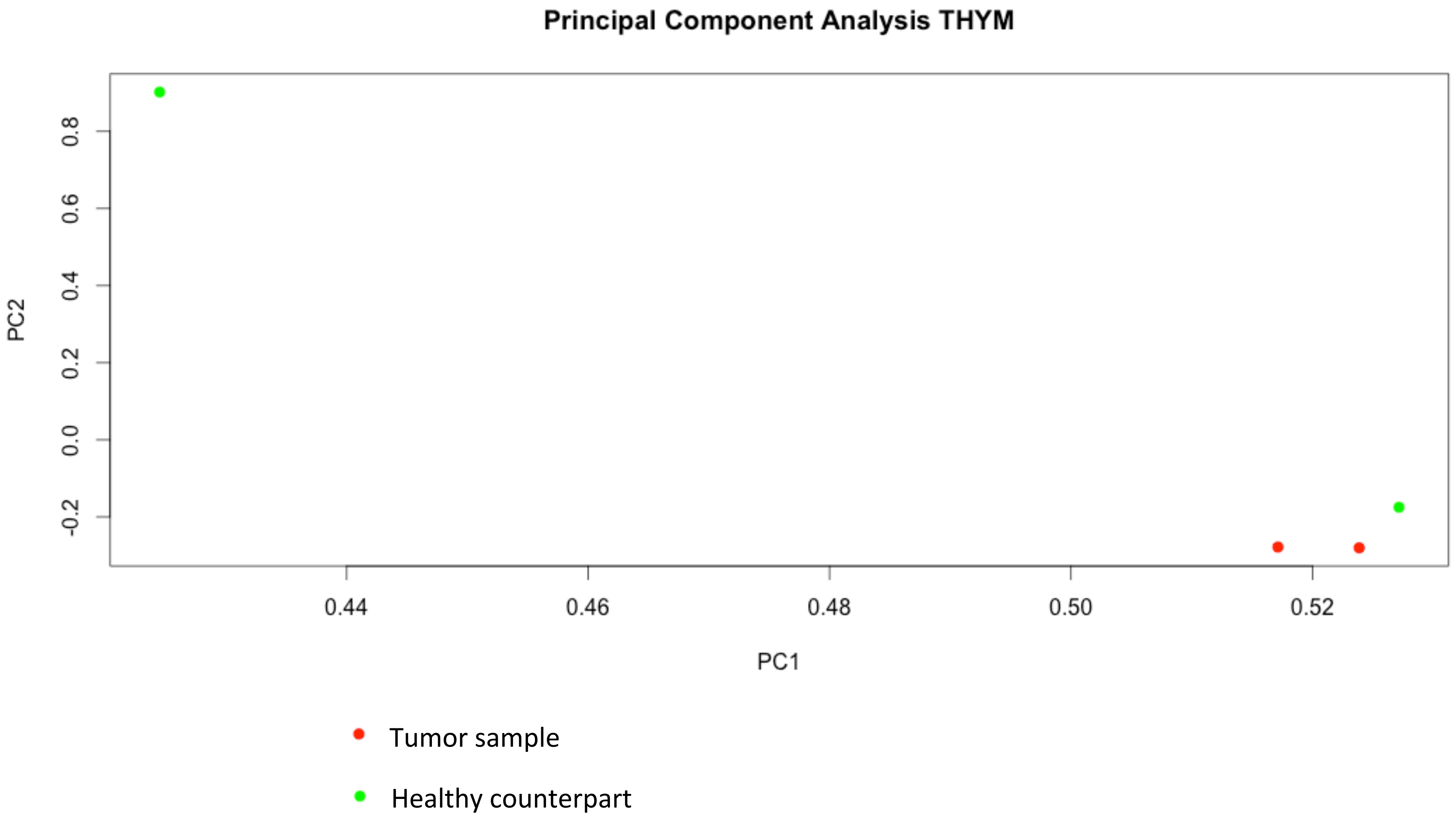
Principal Component Analysis THYM
PC2
0.8
0.6
0.4
0.2
0.0
-0.2
0.44
0.46
0.48
0.50
0.52
PC1
Tumor sample
Healthy counterpart

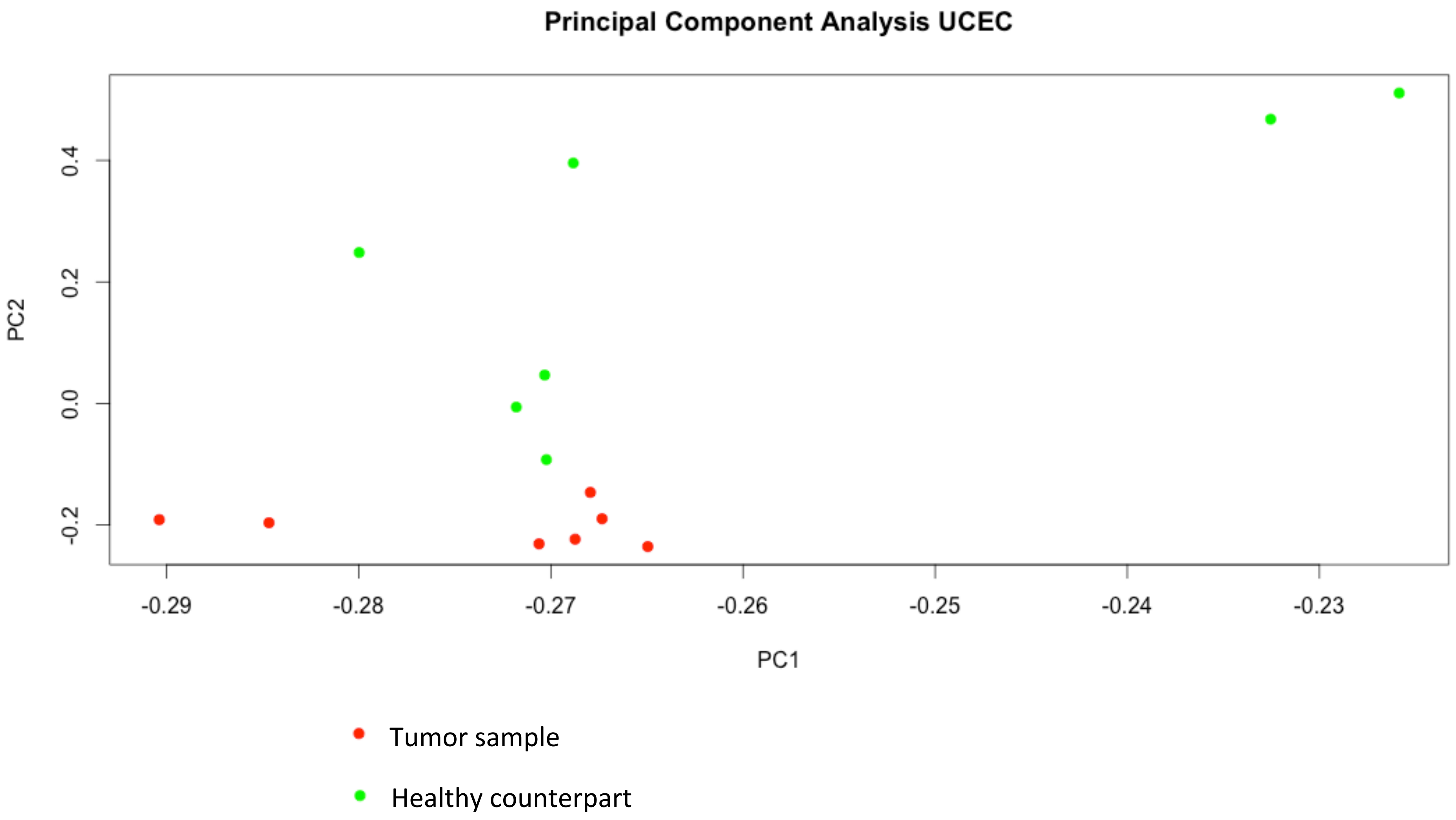
Principal Component Analysis UCEC
PC2
0.4
0.2
0.0
-0.2
-0.29
-0.28
-0.27
-0.26
-0.25
-0.24
-0.23
PC1
Tumor sample
Healthy counterpart
